# Network pharmacology and experimental validation to study the potential mechanism of Tongguanteng injection in regulating apoptosis in osteosarcoma

**DOI:** 10.1186/s12906-024-04354-z

**Published:** 2024-01-31

**Authors:** Lanyi Wei, Jingjing Meng, Danfeng Xiang, Quanjun Yang, Yangyun Zhou, Lingyan Xu, Mengyue Wang, Junjun Chen, Yonglong Han

**Affiliations:** 1 Department of Pharmacy, Shanghai Sixth People’s Hospital Affiliated to Shanghai Jiao Tong University School of Medicine, Shanghai, 200030 China; 2 School of Pharmacy, Shanghai Jiao Tong University, Shanghai, 200240 China

**Keywords:** Tongguanteng injection, Osteosarcoma, Apoptosis, Network pharmacology, Molecular docking

## Abstract

**Objective:**

The main objectives of this study were to identify the active components of Tongguanteng injection (TGT) and investigate the preclinical efficacy and mechanism of TGT on osteosarcoma using a combination of network pharmacology and experimental validation.

**Methods:**

To identify the active constituents and targets of TGT against osteosarcoma using network pharmacology, we constructed a network consisting of an 'active ingredient-disease-target-pathway' and a protein–protein interaction (PPI) network. The target organ network was utilized to investigate the distribution of core targets in tissues. Afterwards, the core targets underwent Gene ontology (GO) functional enrichment and Kyoto Encyclopedia of Genes and Genomes (KEGG) analyses. The binding energy between receptors and ligands was compared using molecular docking. In addition, SwissADME was employed to forecast the pharmacokinetic characteristics of the substances. Finally, real-time polymerase chain reaction (RT-PCR), cell proliferation assay, morphological analysis, apoptosis assay, mitochondrial membrane potential (MMP) detection, and Western blotting were utilized to confirm the potential mechanisms of TGT treatment in osteosarcoma cell lines 143B and SAOS2.

**Results:**

A total of 54 chemical constituents of TGT and 71 targets associated with osteosarcoma were acquired. Through the molecular docking technology, Tenacigenin B, Marsdekoiside, Taraxasterol, Tenacissoside G, Tenacissoside L, and Tenacissoside J were identified as the primary active components of TGT among the various compounds. Analysis of target organs suggests that TGT may play an anti-osteosarcoma role through immune regulation. The GO and KEGG enrichment analysis revealed that TGT could trigger osteosarcoma cell apoptosis by inhibiting the HIF-1 signalling pathway and modulating PD-1 expression and the PD-1 checkpoint pathway in cancer. SwissADME database predicted that Tenacigenin B and Taraxasterol had the best drug-likeness. In vitro studies also demonstrated that TGT suppressed the activity and induced alterations in the morphology of osteosarcoma cells. It decreased MMP levels, triggered apoptosis by increasing Bax expression and Caspase-3 activity, and decreased Bcl-2 expression, thereby exerting an anti-osteosarcoma effect. In the meantime, RT-PCR tests demonstrated that TGT could control immune response against tumors and hinder the proliferation and spread of cancerous cells by impacting the levels of critical factors, including JUN, HSP90AA1, HDAC1, and CDK1.

**Conclusion:**

The study accurately anticipated the active components, targets, and pathways of TGT in the management of osteosarcoma. The molecular mechanism of TGT-induced apoptosis in osteosarcoma cells was demonstrated by in vitro experiments. These results provide theoretical and technical support for TGT as a clinical adjuvant drug for osteosarcoma.

**Supplementary Information:**

The online version contains supplementary material available at 10.1186/s12906-024-04354-z.

## Introduction

Osteosarcoma, the prevalent primary malignant tumor of the bone, tends to manifest in adolescents and children. High genetic and pathological heterogeneity are distinguishing features of this condition [1, 2]. According to research, the 5-year overall survival rate of patients with localized osteosarcoma is between 65 and 75%, while the 5-year survival rate of patients with recurrent and metastatic tumors is only 20% [3]. Osteosarcoma cells usually show highly and locally aggressive growth. The growth and spread of tumors can be suppressed by combining surgical procedures, chemotherapy (including cisplatin, doxorubicin, and methotrexate), and radiotherapy [4, 5]. Nevertheless, conventional therapeutic approaches may not consistently yield the desired outcomes for osteosarcoma, and the development of chemoresistance remains a significant contributor to treatment ineffectiveness [6]. New treatment methods, such as immune-based targeted therapy, can overcome the immunosuppression of the tumor microenvironment by targeting immune checkpoints. Nevertheless, additional clinical investigations are still required because of the considerable diversity among patients with osteosarcoma [7]. Therefore, finding alternative and natural therapies with equal efficacy and fewer adverse effects for osteosarcoma has attracted much attention.

Traditional Chinese medicine (TCM) and natural remedies contain a diverse range of substances that can target multiple molecular pathways, thereby improving the efficacy of cancer therapy and minimizing negative drug responses [8]. Tongguanteng injection (TGT) is primarily composed of soluble compounds derived from the stem of *Marsdenia tenacissima* (Roxb.) Wight et Arn, serving as a TCM formulation, are frequently employed in prescriptions for cancer treatment. It has been approved for the adjunctive therapy of malignant neoplasms, including esophageal cancer, gastric cancer, lung cancer and liver cancer [9–11]. Patients generally tolerate TGT well and it is frequently used in conjunction with chemotherapy medications like cisplatin and paclitaxel, resulting in a notable extension of cancer patients' survival duration [12, 13]. Numerous pieces of evidence indicate that the C21 steroids derived from TGT possess an extraordinary suppressive impact on various cancer cell lines, including A549, Caco-2, HepG2, K562, PC-3, and SACC83. This discovery holds the potential to significantly enhance the well-being of individuals battling cancer [14]. Nevertheless, limited research has been conducted regarding the impact of TGT on osteosarcoma, and its underlying mechanism is yet to be clarified.

Professor Hopkins, a pharmacologist from the University of Dundee in the United Kingdom, introduced the network pharmacology approach to address the intricate nature of TCM. This method aids in the establishment of a comprehensive network connecting components, targets, diseases, and molecular pathways [15, 16]. In addition, molecular docking can predict the binding affinity between ligands and receptors by simulating their interactions. With the integration of bioinformatics technology, network pharmacology has become an essential field capable of forecasting the molecular-level biological mechanism of TCM on diseases, facilitating rapid identification of potential active drugs and targets. Hence, this research first delved into the therapeutic potential of TGT on osteosarcoma through network pharmacology and in vitro experiments. The findings offer valuable backing for future exploration into the therapeutic mechanism of TGT.

## Material and methods

### Network pharmacology analysis

#### Screening of the active ingredients and targets of TGT

The main components of TGT are derived from the medicinal herb Marsdenia tenacissima. The chemical compositions of Marsdenia tenacissima are sourced from the Herb database (http://herb.ac.cn/). This database collects information on TCM ingredients from SymMap, TCMID, TCMSP, and TCM-ID databases. The active ingredient's SMILES structure is obtained from the PubChem (https://pubchem.ncbi.nlm.nih.gov/) database. To predict the potential targets of TGT, the SwissTargetPrediction (https://www.swisstargetprediction.ch/) database is used. The species is defined as "Homo sapiens". A screening condition of "Probability > 0" is applied, and the target proteins are converted to human genes using the Uniprot (https://www.uniprot.org/) database.

#### Construction of protein–protein interaction (PPI) network

The PPI network of interacting genes was constructed using the STRING (https://string-db.org/) database. The species was set to "Homo sapiens", and a minimum interaction score of "high confidence (0.700)" was applied as the threshold for defining the interactions. In this study, the Cytoscape 3.9.0 plugin CytoNCA was employed to calculate three topological properties of each node in the PPI network: betweenness centrality (BC), closeness centrality (CC), and degree centrality (DC). The top 14 targets with values more significant than the median were selected as central targets. Additionally, the MCODE plugin was used to identify densely connected protein clusters within the PPI network based on the evaluation of cluster density. A cluster score of >  = 3 was applied for clustering analysis.

#### Differential gene analysis of the GEO dataset

We obtained transcriptome sequencing series GSE99671 from the GEO (www.ncbi.nlm.nih.gov/geo/) database, which includes gene expression profiles of osteosarcoma and normal bone samples. Using the bioinformatics (https://www.bioinformatics.com.cn/) online tool, we generated a volcano plot of differentially expressed genes (DEGs) and a heatmap of the top 30 differentially expressed genes from the intersection of TGT-related genes and osteosarcoma genes [17]. The volcano map and heatmap were created using the pheatmap and enhancedvolcano R packages. The threshold for identifying DEGs is set as |logFC|> 2 and *P* < 0.05.

#### Construction of osteosarcoma target genes and prediction of potential drug targets

Download osteosarcoma genes from the GeneCards (http://www.genecards.org/) database, DisGeNET (https://www.disgenet.org/) database, and DrugBank (https://go.drugbank.com/) database. Remove duplicate genes to obtain osteosarcoma target genes. Visualize the target genes contained in TGT and the active components of osteosarcoma using Venny 2.1.0 (https://bioinfogp.cnb.csic.es/tools/venny/), and consider the intersected target genes as candidate target genes. Finally, input the overlapping targets and related components into Cytoscape 3.9.0 (https://cytoscape.org/) and filter potential drug targets using the condition "degree >  = 5".

#### Construction of target organ network

The complete distribution and metabolism of TGT in the human body have yet to be fully elucidated. Establishing a target organ network can help predict drug efficacy and adverse reactions, improving drug safety and effectiveness. In this study, we utilized the BioGPS (https://biogps.org) database to obtain gene tissue-specific mRNA expression levels and visualized the data using Cytoscape 3.9.0.

#### Gene ontology (GO) functional enrichment and kyoto encyclopedia of genes and genomes (KEGG) pathway enrichment analysis

The core targets were imported into the DAVID (https://david.ncifcrf.gov) database for GO functional annotation and KEGG pathway enrichment analysis. We retained KEGG entries with a significance level of *P* < 0.01 for analysis, sorted by fold enrichment. GO terms were divided into three categories: Biological Process (BP), Cellular Component (CC), and Molecular Function (MF). Using a bioinformatics platform for visualizing the top ten GO terms based on P-values and KEGG pathways with *P* < 0.01. To better analyze the mechanism of the TGT anti-osteosarcoma effect, we constructed a "TCM—Target Gene—Disease—Pathway" network and visualized it using Cytoscape 3.9.0.

#### Molecular docking

Molecular docking is a virtual screening technique that can predict and simulate the binding mode and affinity between small molecules (ligands) and target molecules (receptors) [18]. Retrieve the 2D structure of active compounds from the PubChem database, import it into ChemBio 3D 17.0 software for MM2 energy minimization optimization, and save it in mol2 format. Obtain the protein crystal structure of the core target for osteosarcoma in TGT from the PDB (https://www.rcsb.org/) database. Use PyMOL 2.5.4 software to remove water molecules and residual ligands and save the structure in pdb format. Then, preprocess the receptor protein by adding hydrogens using Autodock Tools 1.5.7 software and save the processed file in pdbqt format. Use AutoDockTools software for molecular docking between the active drug compounds and the receptor protein, establish the docking pocket, and calculate the binding energy. The relevant strands were identified within the protein, and the active site residues were labelled within a 4 Å radius of the ligand. Visualize the docking results using PyMOL 2.5.4 software.

#### Pharmacokinetic evaluation

The SMILES of the active ingredient were collected from the PubChem database, and the physicochemical properties, pharmacokinetic parameters, and drug-likeness of the compound were assessed using the online website SwissADME (http://www.swissadme.ch/).

### Experimental validation

#### Chemicals and reagents

TGT was the water extract from the stem of Marsdenia tenacissima (Roxb.) Wight et Arn. Each milliliter (mL) injection contains 1 g dry weight of herb powder. TGT was purchased from Nanjing Sanhome Pharmaceutical Co.LTD. The stem of Marsdenia tenacissima (Roxb.) Wight et Arn. was collected from Yunnan, China. A voucher specimen (200,907-T009-05) was deposited in the herbarium of San Home Pharmaceutical Co. LTD and was identified by Professor De-Kang Wu from Nanjing University of Chinese Medicine. The Marsdenia tenacissima (Roxb.) Wight et Arn. stem was extracted as previously described [19]. The TGT manufacturing process was prepared following the standard of the 2020 edition of Pharmacopoeia of The People's Republic of China (Ch.P.) and China Food and Drug Administration (WS-10630(ZD-0630)-2002-2013Z-2019). The main components detected by fingerprint spectra were tetrahydrocurcumin, neochlorogenic acid, caffeic acid, chlorogenic acid, syringic acid, cryptochlorogenic acid, 4-coumaric acid, and sinapic acid. The amount of total steroidal saponins (calculated as Tenacigenoside A (C_35_H_56_O_12_)) should be not less than 8.0 mg/mL, which was determined using high-performance liquid chromatography.

Dulbecco's Modified Eagle's medium (DMEM) and fetal bovine serum (FBS) were provided by Gibco (New York, USA). The penicillin/streptomycin solution and ProtLytic phosphatase inhibitor mixture were obtained from NCM Biotech (Suzhou, China). Cell Counting Kit-8 (CCK-8), Z-VAD-FMK, Rhodamine 123, BCA kit, and Caspase-3 Activity Assay Kit were acquired from Beyotime (Shanghai, China). RIPA lysis buffer was purchased from Solarbio (Beijing, China). 3-Methyladenine (3-MA) was purchased from MedChemExpress (New Jersey, USA). APC Annexin V/7-AAD Apoptosis Detection Kit was purchased from Biolegend (San Diego, USA). EZ-press RNA Purification Kit and the Color Reverse Transcription Kit with gDNA Remover were obtained from EZBioscience (Roseville, USA). A protein-free blocking buffer and a chemiluminescence detection kit were purchased from Epizyme (Shanghai, China).

#### Cell culture and treatment

The osteosarcoma cell lines 143B and SAOS2, derived from human sources, were acquired from the Cell Bank of the Committee on Preservation of Typical Cultures, Chinese Academy of Sciences. The cells were grown in a high-glucose DMEM medium containing 10% fetal bovine serum and 1% penicillin/streptomycin solution. The cells were cultured nonstop at 37 °C with a 5% concentration of CO_2_ in a specialized incubator for cell culture. 143B and SAOS2 cells in the logarithmic growth phase were utilized for the following trials. For the subsequent experiments, the 96- or 6-well plates were used to seed the cells, which were then cultured with varying amounts of TGT (0, 12.5, 25, 50, 75, 100, and 200 mg/mL) for durations of 12, 24, and 48 h.

#### Cell viability assay and morphological observation

Utilize the Cell Counting Kit-8 kit for assessing cell viability. Inoculate 100 μL of processed 143B and SAOS2 cells into aseptic 96-well plates (1.0 × 10^4^ cells/well) and incubate them in a CO_2_ incubator at 37 °C with 5% CO_2_ and full humidity. Start drug treatment at concentrations of 0, 12.5, 25, 50, 100, and 200 mg/mL after 24 h of seeding the cells. Following 12, 24, and 48 h of therapy, discard the liquid above and introduce 100 μL of DMEM solution with 10% CCK-8 into every well. Place in the incubator for another hour, then use an EPOCH multi-function microplate reader (BioTek, USA) to determine the absorbance at 450 nm. Construct a dose–response curve and calculate the IC_50_ value of TGT against osteosarcoma inhibition. Expose the cells to 50 and 75 mg/mL concentrations, either with or without the autophagy inhibitor 3-MA and the apoptosis inhibitor Z-VAD-FMK, for 48 h under identical circumstances. Subsequently, reevaluate the absorbance. Using an ECLIPSE TS2 inverted fluorescence microscope (Nikon, Japan), capture images of the cells treated with TGT (0, 25, 50, and 75 mg/mL) for 48 h to observe their morphological changes.

#### Flow cytometry

Analyze cell apoptosis through flow cytometry using the APC Annexin V/7-AAD Apoptosis Detection Kit. The cells that are growing exponentially are placed in a 6-well plate with a density of 1.0 × 10^6^ cells per well. The TGT concentrations of 25, 50, and 75 mg/mL are administered to the experimental groups, whereas the control group is treated with an entire culture medium. Gather the suspended and adherent cells after 48 h. Rinse the cells twice using PBS and suspend them in 100 μL of Annexin V binding buffer. Combine the cell suspension with 5 μL of APC Annexin V and 5 μL of 7-AAD viability dye, ensuring a gentle mixing. Place the cells in an incubator at 25 °C, ensuring they are shielded from light, and leave them for 15 min. Mix well after adding 400 μL of Annexin V binding buffer. Use a CytoFLEX flow cytometer (BECKMAN COULTER, USA) for cell analysis to ascertain the proportion of cells undergoing apoptosis. Process the data using CytExpert software.

#### Mitochondrial membrane potential (MMP) analysis

The Rhodamine 123 MMP Assay Kit is utilized to evaluate the effect of TGT on osteosarcoma cell apoptosis. Rhodamine 123 is used as a fluorescent probe in this assay. Following TGT treatment, the 143B and SAOS2 cells are exposed to a Rhodamine 123 staining solution in a light-protected environment and kept in a cell culture incubator at 37 °C for 30 min. After discarding the supernatant, the cells are washed two times with a culture medium and observed using a fluorescence microscope.

#### Caspase-3 activity assay

The kit for measuring Caspase-3 activity, known as the Caspase-3 Activity Assay Kit, is employed to assess the impact of TGT on the activity of Caspase-3 in 143B and SAOS2 cells. The osteosarcoma cells undergo lysis following a 48-h TGT treatment (0, 25, 50, and 75 mg/mL). Subsequently, they are mixed with the detection buffer and 2 mM Ac-DEVD-pNA, resulting in a solution of 100 μL in total volume. Following incubation at a temperature of 37 °C inside a CO_2_ incubator for 24 h, the OD of the specimens is assessed using a microplate reader with a wavelength of 405 nm.

#### Western blotting analysis

A suitable quantity of RIPA buffer with a combination of protease inhibitor mixture and ProtLytic phosphatase inhibitor mixture was added for protein extraction to lyse osteosarcoma cells. The BCA kit was utilized to ascertain the protein concentration. The protein samples underwent separation using 12.5% SDS-PAGE and were subsequently transferred to a PVDF membrane (0.45 μm, Millipore Corporation, USA). PVDF membranes were cropped along the molecular weight marker region to separate the target protein. Quantitative comparisons of different protein samples were also guaranteed to come from the same PVDF membrane. After incubation in 1 × protein-free blocking buffer at room temperature for 30 min, the samples were exposed to primary antibodies overnight at 4 °C. The next day, the samples were exposed to secondary antibodies at ambient temperature for 1 h. Following three rounds of washing with TBST for 2 min each, the protein bands were identified using the Omni-ecl Ultra-Sensitive Chemiluminescence Detection Kit in the ImageQuant LAS 4000 mini visualizer (GE Healthcare Bio-Sciences AB). ImageJ software was used to analyze the grayscale values of protein bands, and each experiment was repeated three times. The primary antibodies utilized were the following: anti-Bax (A19684, ABclonal, China), anti-Bcl-2 (A19693, ABclonal, China), anti-PCNA (13110 T, Cell Signaling Technology, USA), and anti-Actin (4970 T, Cell Signaling Technology, USA).

#### Real-time polymerase chain reaction (RT-PCR) analysis

Total RNA is extracted from the treated cells using the EZ-press RNA Purification Kit. Afterwards, the RNA is converted into cDNA using the Color Reverse Transcription Kit alongside the gDNA Remover. To prepare a qPCR reaction system of 10 μl, mix the cDNA with 2 × Color SYBR Green qPCR Master Mix (ROX1 plus), primers, and ddH2O. Four replicates are set for each sample. The cycling parameters are as follows: 95 °C for 5 min, 95 °C for 10 s, 60 °C for 30 s, and 72 °C for 30 s. The relative expression levels are determined using the 2^-△△Ct technique. Supplementary Table 1 contains the listed primer sequences.

### Statistical analysis

Statistical analysis was performed using GraphPad Prism 9.0 software. The experimental data were expressed as mean $\pm$ standard deviation. One-way Analysis of Variance (ANOVA) was conducted to compare multiple groups with one independent variable. A P value less than 0.05 was considered statistically significant.

## Results

### Screening active ingredients and targets of TGT for osteosarcoma

**Fig. 1:**
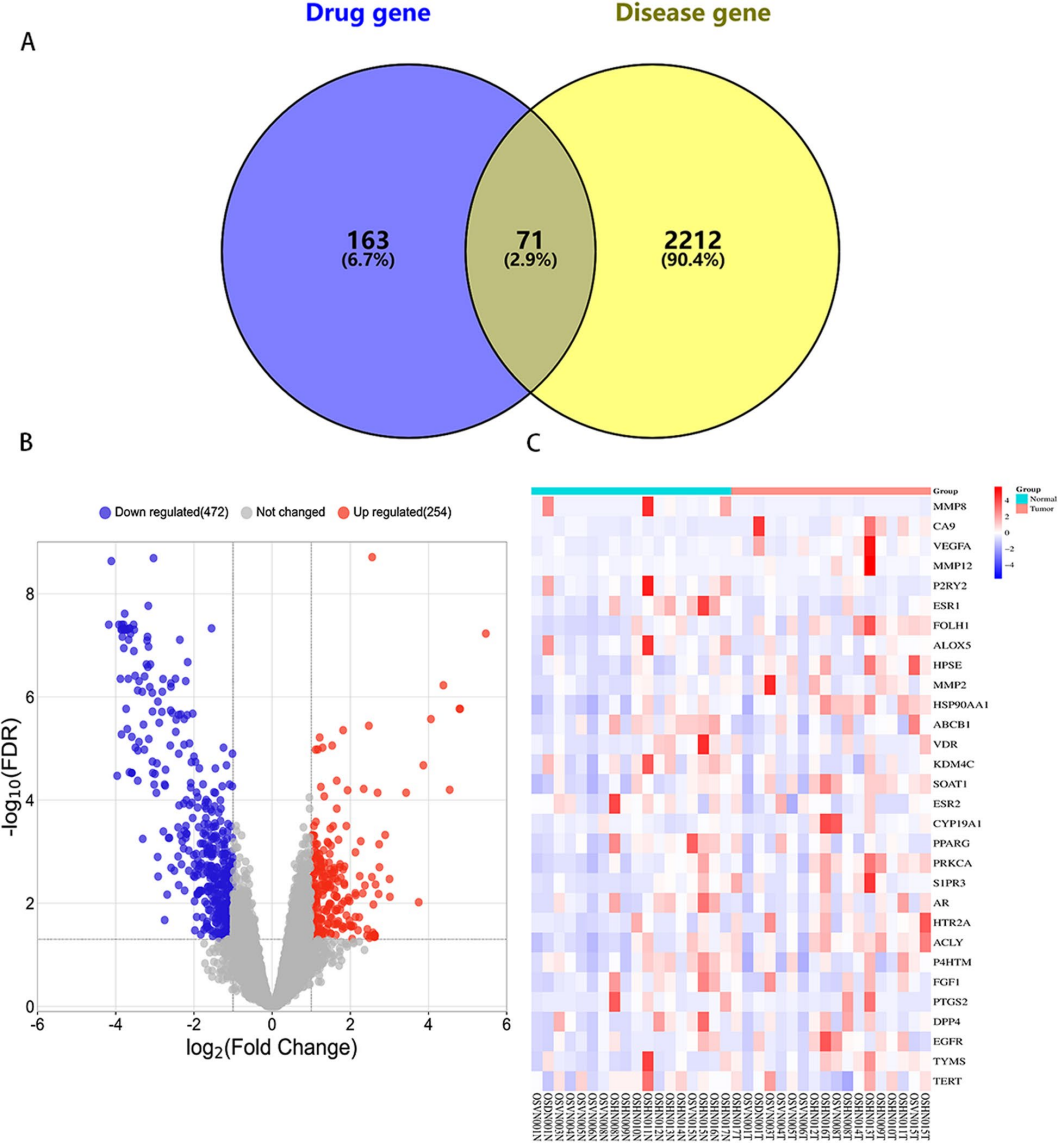
Screening for common targets between TGT and osteosarcoma. **A** Venn diagram of the target genes of TGT's active ingredients and the target genes of osteosarcoma. **B** Volcano plot of the differentially expressed genes in the GSE99671 series. Downregulated genes are represented in blue, while upregulated genes are represented in red. **C** Heatmap showing the top 30 differentially expressed genes among the 71 intersecting targets in the GSE99671 series

First, the 54 main active ingredients of TGT were collected from the Herb database. By removing duplicate targets, 234 potential protein targets were obtained for the active ingredients from the SwissTargetPrediction database. By searching the GeneCards and DisGeNET databases, resulting in 2,283 osteosarcoma-related target genes were identified. Using Venny 2.1.0, 71 overlapping genes were selected as candidate targets by taking the intersection of the protein targets of TGT and osteosarcoma targets (Fig. 1A). Based on the GSE99671 series, 726 differentially expressed genes were identified in osteosarcoma. Among them, 254 genes were significantly upregulated. In comparison, 472 genes were downregulated (Fig. 1B). According to the GSE99671 series, the expression heat map of 30 genes with significant differential expression was drawn (Fig. 1C). Additionally, the Cytoscape 3.9.0 was utilized to filter nodes based on the threshold "degree > 5". As shown in Table 1 and Table 2, 26 core TGT components and 50 associated targets for osteosarcoma were identified after removing non-overlapping targets.

**Table 1:** The 26 essential ingredients of TGT for osteosarcoma

Herb ID	Active ingredient	Degree	Betweenness	Closeness
HBIN023844	dihydroconduritol	9	1787.896132	0.138064516
HBIN024399	D-oleandrose	8	471.9776639	0.132589839
HBIN034512	marsdekoiside	8	1286.289284	0.127532777
HBIN045869	tenacigenin B	8	1423.285729	0.13425345
HBIN045881	tenacissoside G	8	1576.932115	0.138064516
HBIN023530	D-glucose	7	226.6314494	0.130647131
HBIN042735	saccharose	7	226.6314494	0.130647131
HBIN045530	taraxasterol	7	648.9497331	0.130647131
HBIN045873	tenacissoside j	7	354.3372835	0.130647131
HBIN045886	tenacissoside L	7	226.6314494	0.130647131
HBIN009337	3-O-methyl-6-deoxy-D-allose	6	849.5495275	0.123699422
HBIN018278	beta-sitosterol	6	169.506258	0.124854142
HBIN022771	daucosterol	6	857.1288797	0.119021135
HBIN033753	Lupeol acetate	6	1064.686759	0.136305732
HBIN035353	methyl palmitate	6	836.5282732	0.133250311
HBIN044918	stigmasterol	6	169.506258	0.124854142
HBIN045879	tenacissoside E	6	81.25440177	0.120359955
HBIN045883	tenacissoside I	6	1004.075542	0.131288344
HBIN045885	tenacissoside K	6	181.7580147	0.130328867
HBIN021338	conduritol	5	534.8716142	0.122565865
HBIN022833	D-cymarose	5	851.3376638	0.137355584
HBIN024358	docosanoic acid	5	865.5726191	0.136653895
HBIN029047	hentriacontane	5	631.0727867	0.118493909
HBIN045876	tenacissoside B	5	599.9311288	0.130328867
HBIN045880	tenacissoside F	5	32.7600876	0.119553073
HBIN045887	tenacissoside M	5	32.7600876	0.119553073

**Table 2:** The fifty targets of TGT against osteosarcoma

Num	Target	Degree	Num	Target	Degree	Num	Target	Degree
1	EGFR	74	18	RAF1	26	35	CXCR1	14
2	VEGFA	70	19	TOP1	26	36	VDR	14
3	JUN	58	20	TYMS	26	37	POLA1	14
4	STAT3	56	21	MET	24	38	FOLH1	14
5	HSP90AA1	54	22	PRKCA	22	39	DPP4	12
6	ESR1	50	23	CYP19A1	22	40	POLD1	12
7	PTGS2	48	24	FLT1	20	41	KDM4C	10
8	AR	42	25	CXCR2	18	42	HMGCR	10
9	KDR	38	26	ESR2	18	43	HSD11B1	8
10	CDK1	36	27	AURKB	18	44	HSD11B2	8
11	FGF2	36	28	BRD4	18	45	MMP12	6
12	HDAC1	34	29	AURKA	18	46	PFKFB3	6
13	NR3C1	34	30	LGALS3	16	47	P2RY2	6
14	IL2	32	31	MMP8	16	48	NR1H3	6
15	MMP2	32	32	EIF4E	16	49	KDM2A	6
16	CREBBP	30	33	CDC25A	16	50	DTYMK	6
17	ABCB1	30	34	FGF1	14			

### Construction of "active ingredients-disease-targets-pathways" network and PPI network

**Fig. 2:**
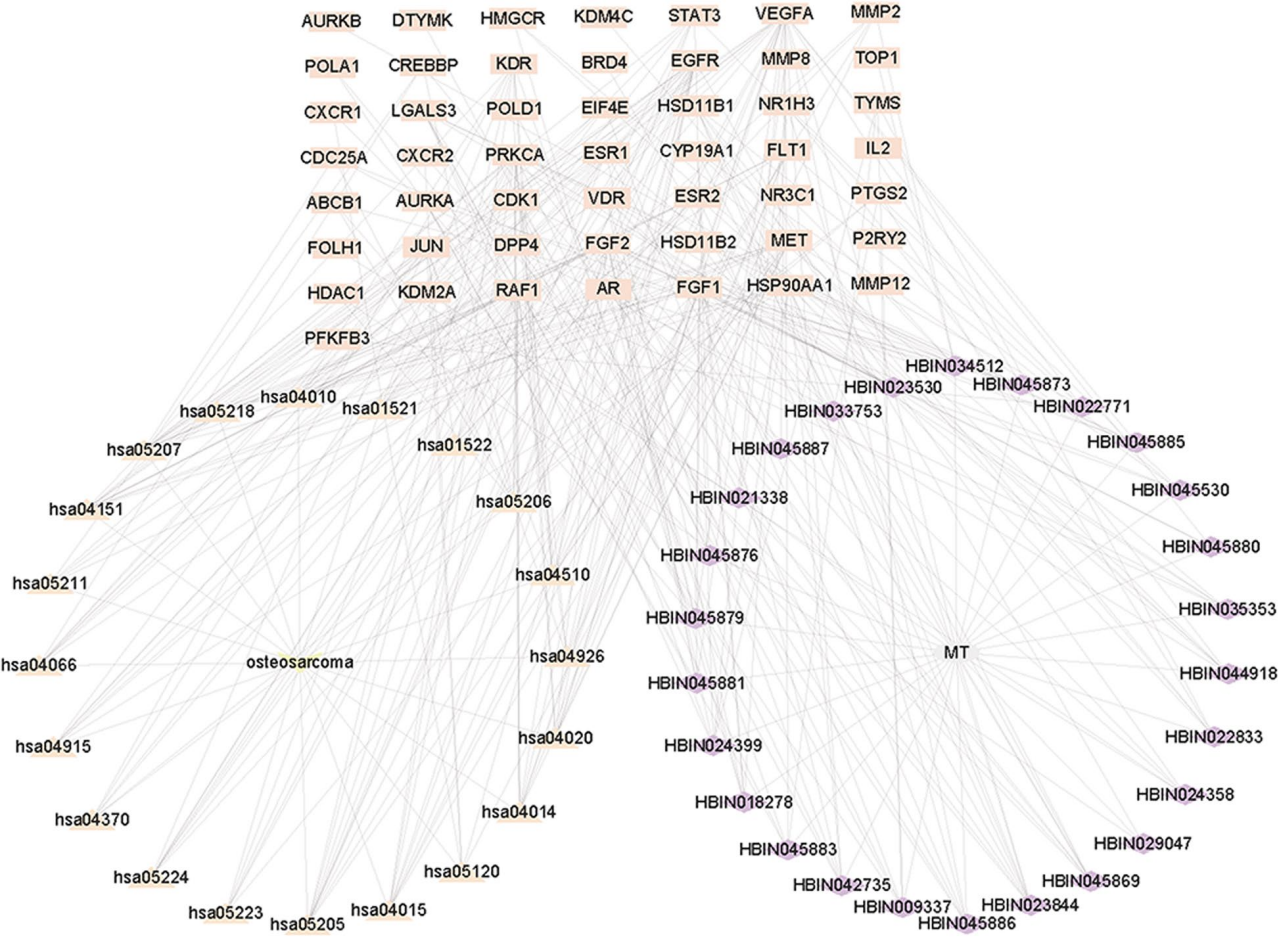
The "active ingredients-disease-targets-pathways" diagram illustrating TGT for treating osteosarcoma. Rectangular nodes represent targets, triangular nodes represent pathways, and diamond nodes represent compounds

We have constructed a network diagram titled "active ingredients-disease-targets-pathways" by combining 26 active ingredients, 50 target proteins, and the top 20 pathways enriched with the lowest P values for the targets (Fig. 2). The visualization was created using Cytoscape 3.9.0.

**Fig. 3:**
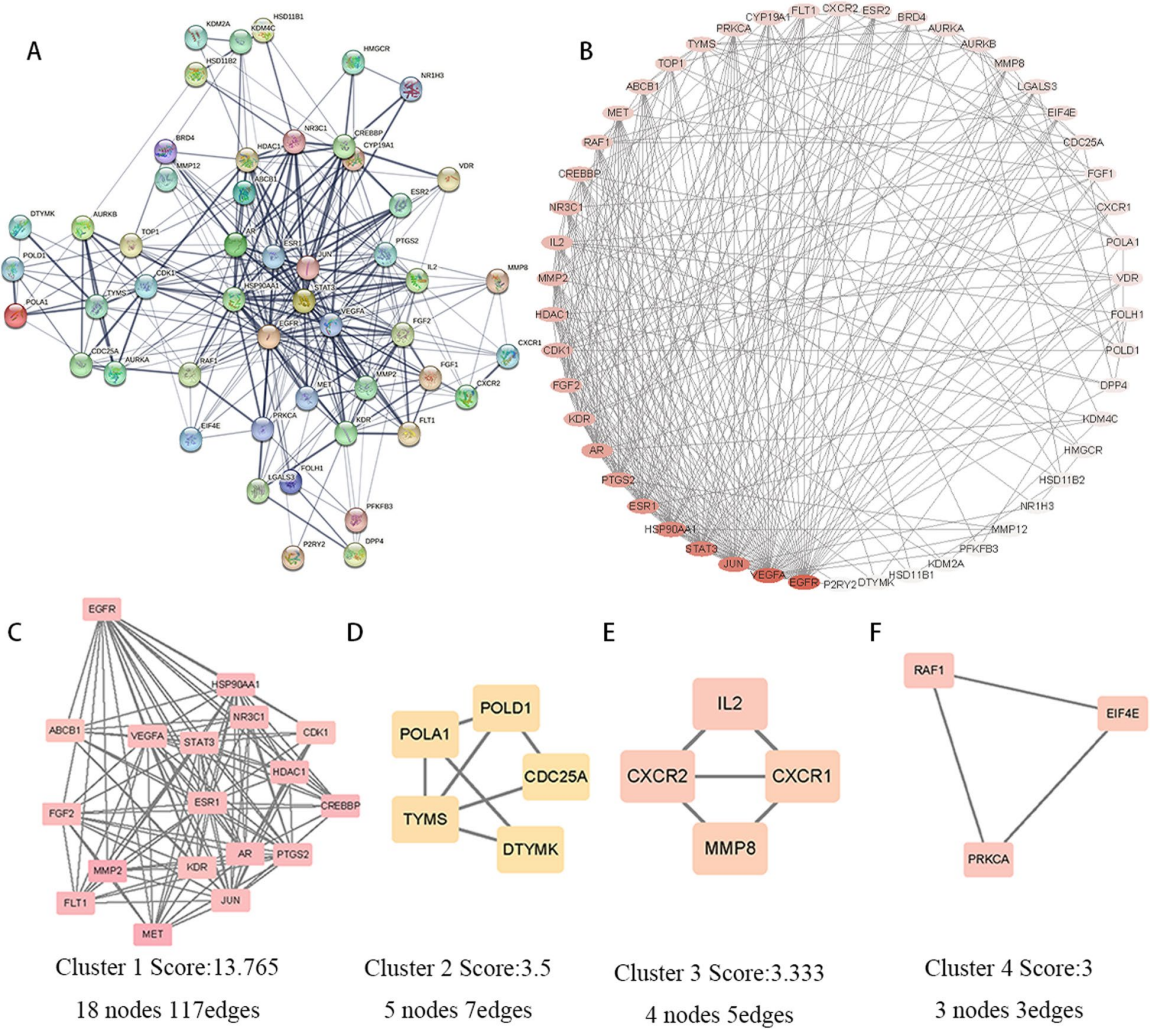
The PPI network of TGT for treatment of osteosarcoma. **A** The PPI network is constructed using the String database. **B** The PPI network was visualized using Cytoscape software. The color intensity represents the degree values of the targets, with darker colors indicating higher degrees. **C**-**F** The PPI network was subjected to cluster analysis using the MCODE plugin

The relevant target proteins were inputted into the STRING database. In the analysis, we set the minimum required interaction score to 0.7. Figure 3A displays the PPI network of target interactions. The PPI network consists of 50 nodes and 301 edges, with an average node degree of 12 and an average local clustering coefficient of 0.664. Visualize the interactions between TGT and osteosarcoma target proteins using Cytoscape 3.9.0. Colors were assigned to the nodes, with darker colors indicating higher degrees (Fig. 3B). We identified 50 nodes, with an average node degree of 12, an average node CC of 0.53, and an average node BC of 0.02. We defined hub genes as those with average values above the mean for degree, CC, and BC parameters (Table 3). Subsequently, we performed cluster analysis using the MCODE plugin to group tightly connected targets into four protein clusters (Fig. 3C-F). The final network results indicate that the 14 core targets of the 26 active ingredients in the herbal injection may be crucial for inhibiting osteosarcoma.

**Table 3:** Information of the 14 critical targets for treating osteosarcoma with TGT

Number	Protein name	Gene name	Uniprot ID	Degree	Betweenness	Closeness
1	Epidermal growth factor receptor	EGFR	P00533	36	0.15100513	0.79032258
2	Vascular endothelial growth factor A	VEGFA	P15692	35	0.15552886	0.77777778
3	Transcription factor Jun	JUN	P05412	29	0.07098548	0.71014493
4	Signal transducer and activator of transcription 3	STAT3	P40763	28	0.04977055	0.7
5	Heat shock protein HSP 90-α	HSP90AA1	P07900	26	0.0566902	0.68055556
6	Estrogen receptor	ESR1	P03372	24	0.03879833	0.66216216
7	Prostaglandin/H synthase 2	PTGS2	P35354	22	0.02259439	0.62025316
8	Androgen receptor	AR	P10275	21	0.03327999	0.63636364
9	Histone deacetylase 1	HDAC1	Q13547	17	0.03491079	0.60493827
10	Cyclin-dependent kinase 1	CDK1	P06493	17	0.06077285	0.59036145
11	Glucocorticoid receptor	NR3C1	P04150	16	0.0463535	0.57647059
12	CREB-binding protein	CREBBP	Q92793	15	0.03937816	0.56976744
13	DNA topoisomerase 1	TOP1	P11387	12	0.02337865	0.55681818
14	Thymidylate synthase	TYMS	P04818	12	0.0278047	0.54444444

### Construction of target-organ network

**Fig. 4:**
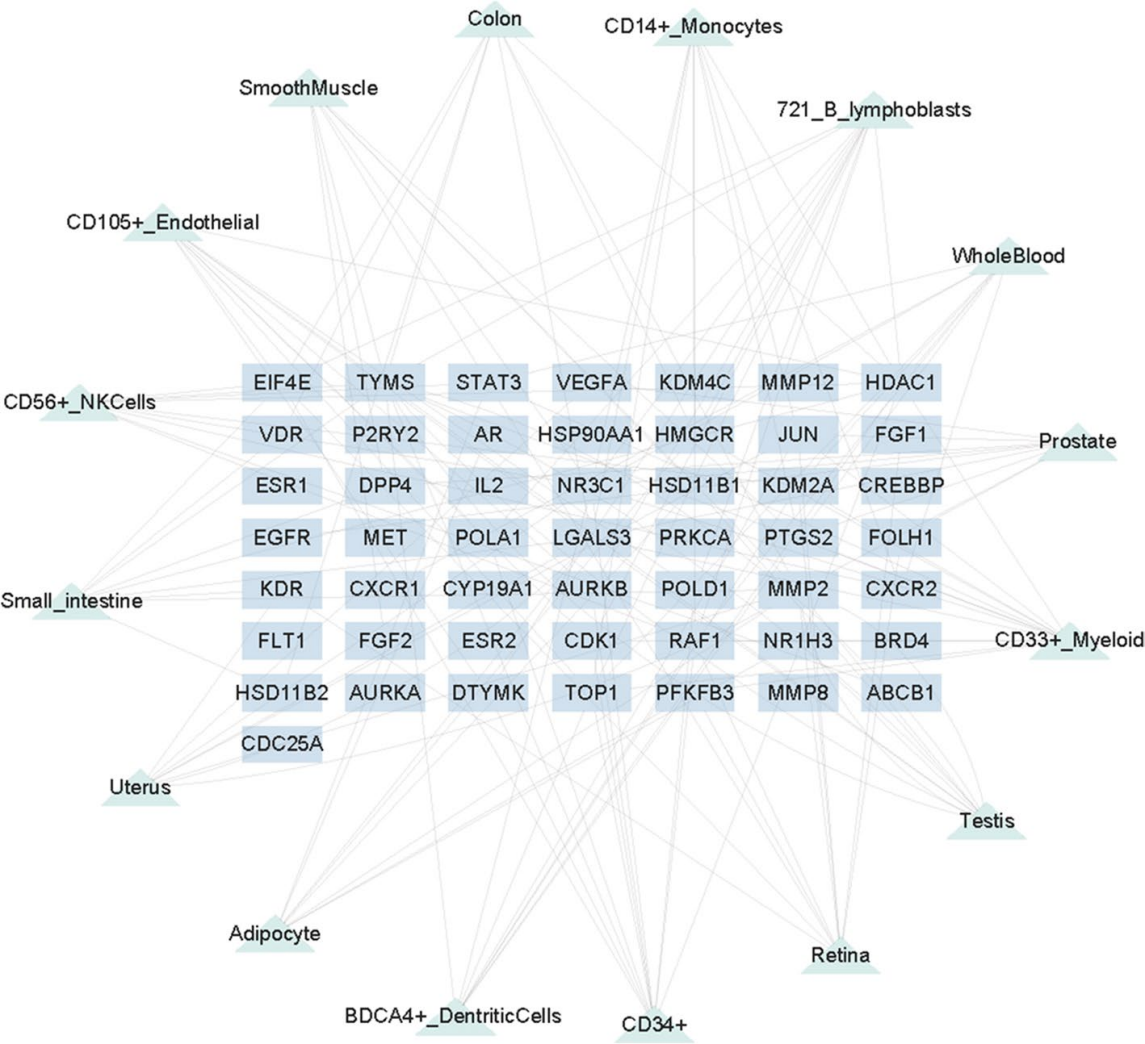
A network was constructed linking the target proteins of TGT with 16 target organs

To explore the possible tissue distribution of TGT, we evaluated the mRNA levels of 50 target genes of TGT in different organs. Among them, 20 target proteins showed relatively high expression in immune organs, including CD33 + _Myeloid (13 targets), 721_B_lymphoblasts (12 targets), CD34 + (11 targets), CD105 + _Endothelial (10 targets), CD14 + _Monocytes (9 targets), and CD56 + _NK Cells (9 targets) (Fig. 4). Additionally, relatively high expression of these target proteins was also observed in multiple tissues such as colon, whole blood, prostate, testis, retina (Supplementary Table 2). Therefore, we hypothesized that TGT may exert its anti-osteosarcoma effect by activating the systemic immune system and coordinating the functions of various body organs.

### GO functional and KEGG pathway enrichment analysis

**Fig. 5:**
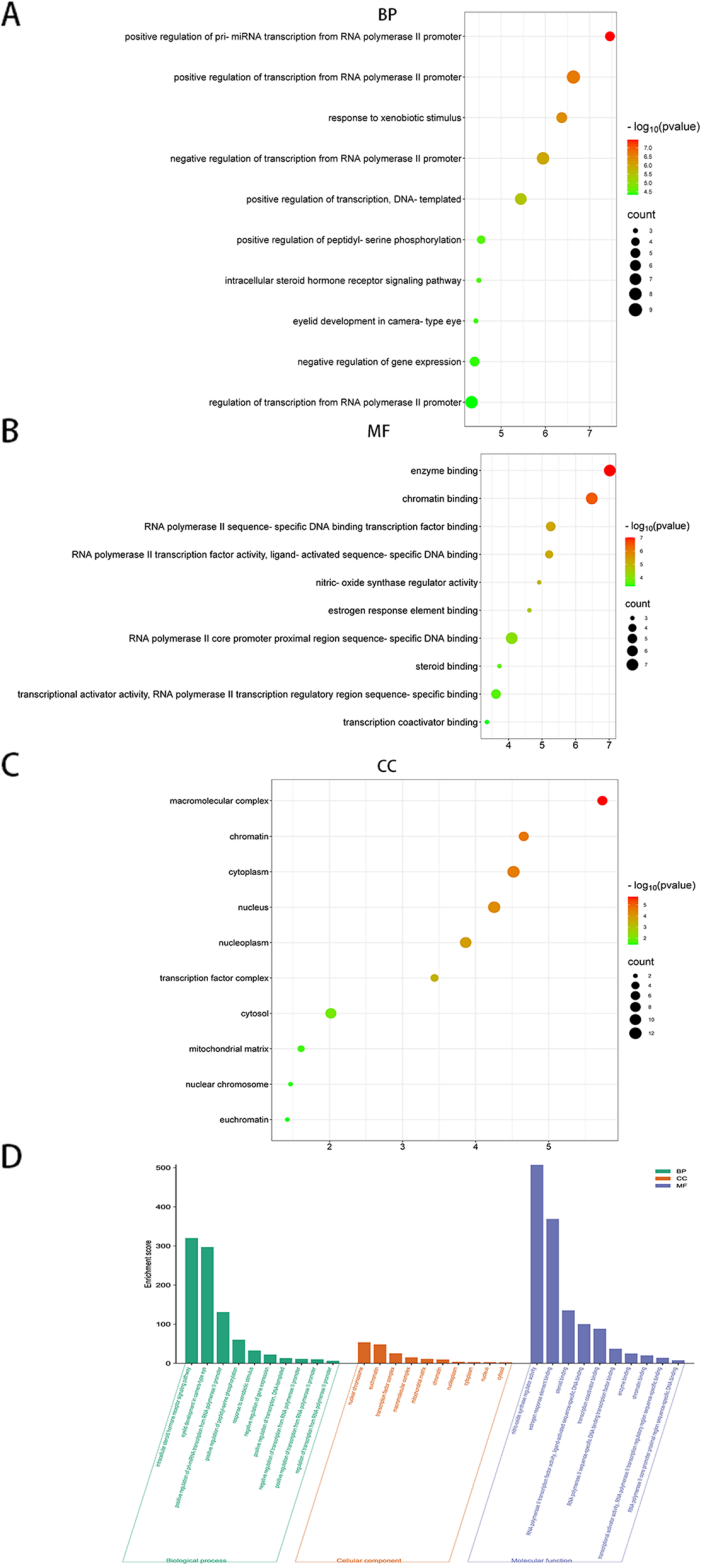
The bubble diagram of GO pathways of candidate genes

**Fig. 6:**
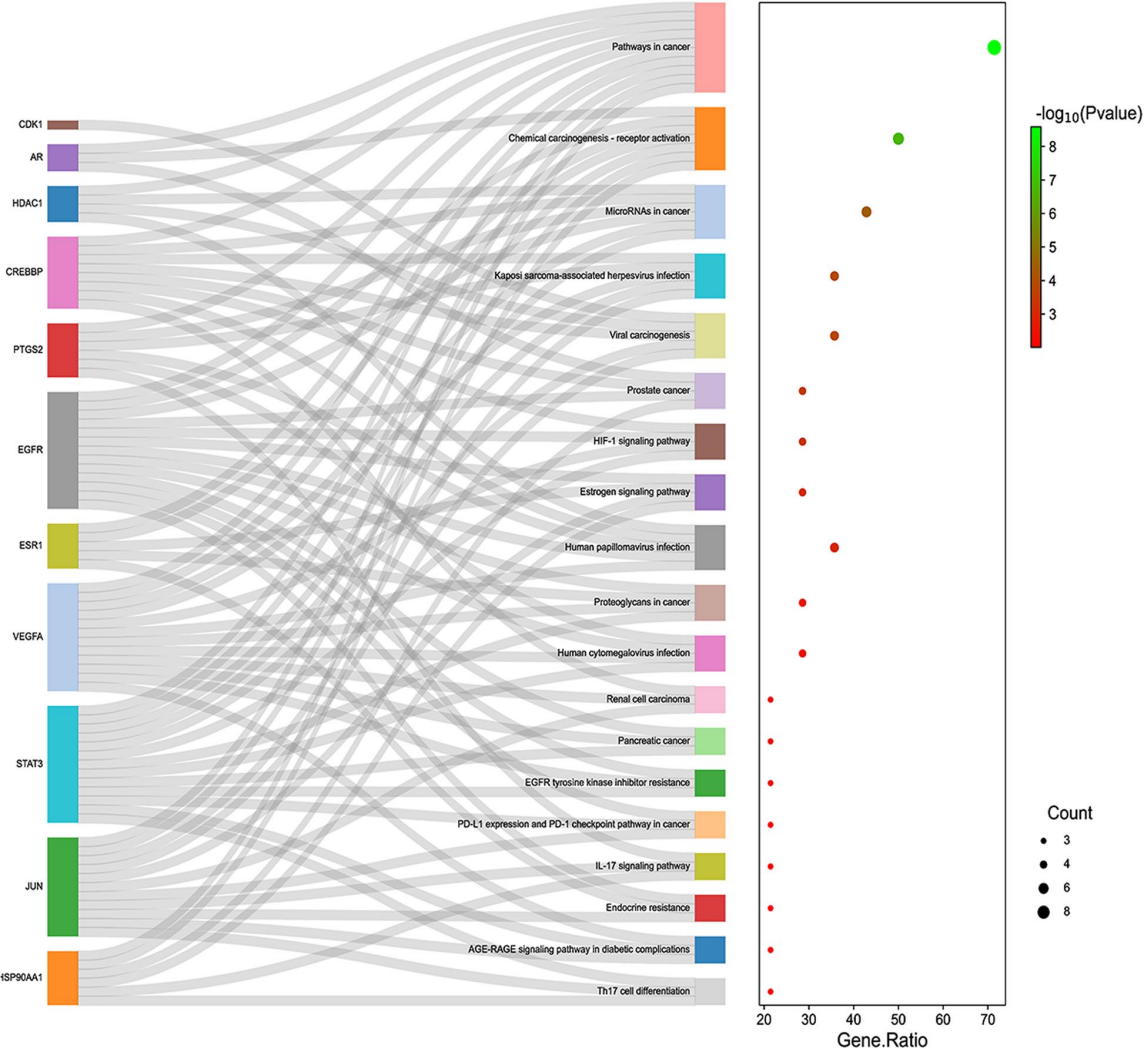
Sankey diagram of KEGG pathways of candidate genes. The rectangles on the left represent target genes, while the rectangles on the right represent pathways

For the 14 candidate genes selected through PPI screening, including EGFR, VEGFA, JUN, STAT3, HSP90AA1, ESR1, PTGS2, AR, HDAC1, CDK1, NR3C1, CREBBP, TOP1, and TYMS, we conducted functional and pathway enrichment analysis using the DAVID database. The top 10 enriched BP, CC, and MF were selected based on their p-values. Bubble and bar plots were generated (Fig. 5). The GO analysis revealed that the enriched BP terms were mainly related to the regulation of intracellular steroid hormone receptor signaling pathway, positive regulation of peptidyl-serine phosphorylation, response to xenobiotic stimulus, and negative regulation of gene expression. The enriched CC terms were primarily associated with nuclear chromosomes, euchromatin, transcription factor complex, and macromolecular complex. The enriched MF terms included nitric-oxide synthase regulator activity, estrogen response element binding, steroid binding, RNA polymerase II transcription factor activity, and ligand-activated sequence-specific DNA binding. Furthermore, KEGG pathway enrichment analysis was performed using the DAVID database, and a total of 19 significant pathways (*P* < 0.01) were identified (Table 4). The Sankey diagram (Fig. 6) was created based on the sorted fold enrichment values. The identified pathways included the HIF-1 signaling pathway (hsa04066), PD-L1 expression and PD-1 checkpoint pathway (hsa05235), and various cancer-related pathways.

**Table 4:** The information of the 19 KEGG pathways obtained using the DAVID database

ID	Term	Gene Ratio	*P* value	Count
hsa05200	Pathways in cancer	71.43%	2.61E-09	10
hsa05207	Chemical carcinogenesis—receptor activation	50.00%	1.87E-07	7
hsa05206	MicroRNAs in cancer	42.86%	4.09E-05	6
hsa05167	Kaposi sarcoma-associated herpesvirus infection	35.71%	1.15E-04	5
hsa05203	Viral carcinogenesis	35.71%	1.39E-04	5
hsa05215	Prostate cancer	28.57%	2.98E-04	4
hsa04066	HIF-1 signaling pathway	28.57%	4.20E-04	4
hsa04915	Estrogen signaling pathway	28.57%	8.37E-04	4
hsa05165	Human papillomavirus infection	35.71%	8.85E-04	5
hsa05205	Proteoglycans in cancer	28.57%	0.00262	4
hsa05163	Human cytomegalovirus infection	28.57%	0.003413	4
hsa05211	Renal cell carcinoma	21.43%	0.0041	3
hsa05212	Pancreatic cancer	21.43%	0.004953	3
hsa01521	EGFR tyrosine kinase inhibitor resistance	21.43%	0.005342	3
hsa05235	PD-L1 expression and PD-1 checkpoint pathway in cancer	21.43%	0.006737	3
hsa04657	IL-17 signaling pathway	21.43%	0.00749	3
hsa01522	Endocrine resistance	21.43%	0.008119	3
hsa04933	AGE-RAGE signaling pathway in diabetic complications	21.43%	0.008442	3
hsa04659	Th17 cell differentiation	21.43%	0.009792	3

### Molecular docking results analysis

**Fig. 7:**
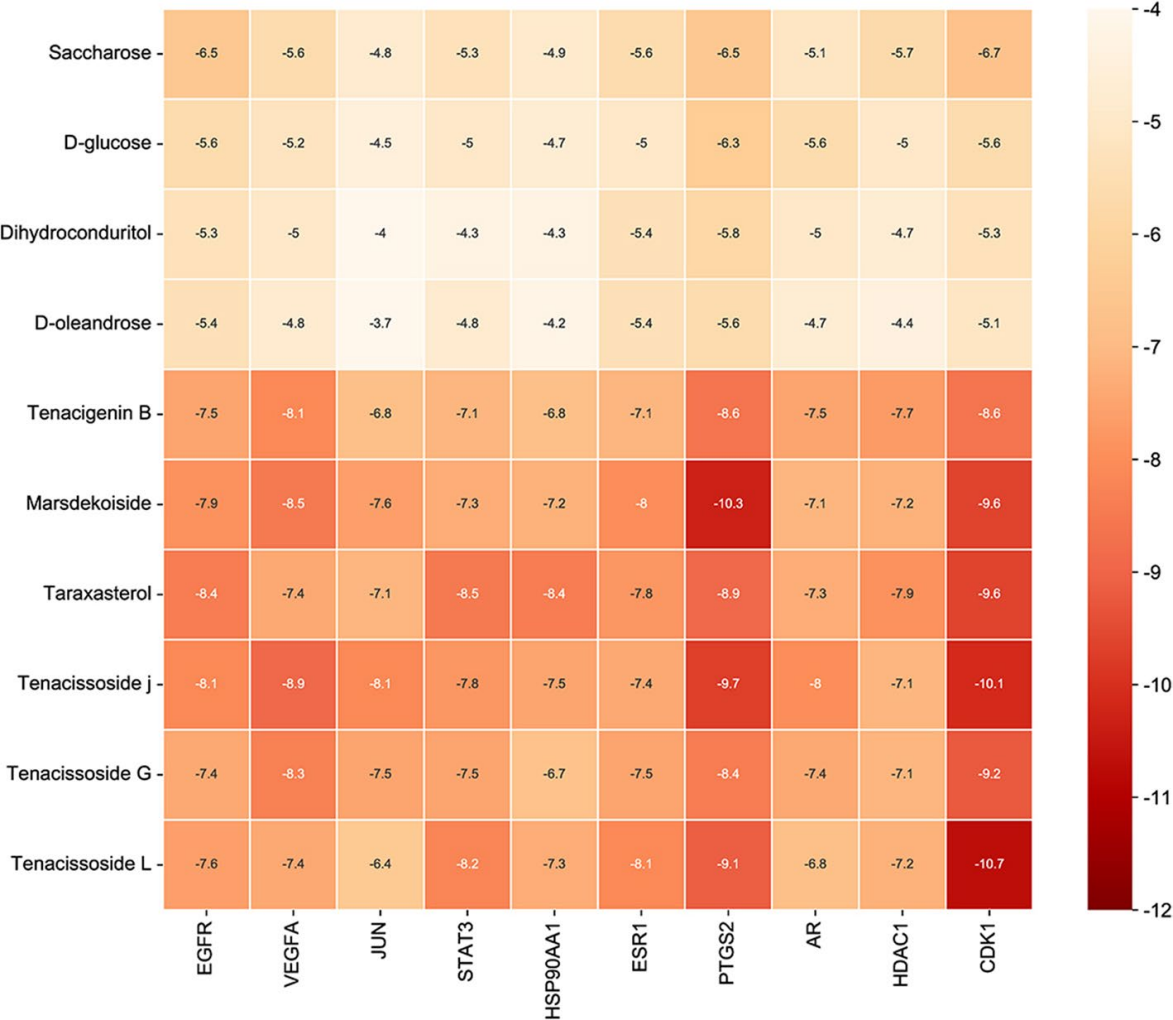
Heatmap of binding energies (kcal/mol) between ligands and receptors

**Fig. 8:**
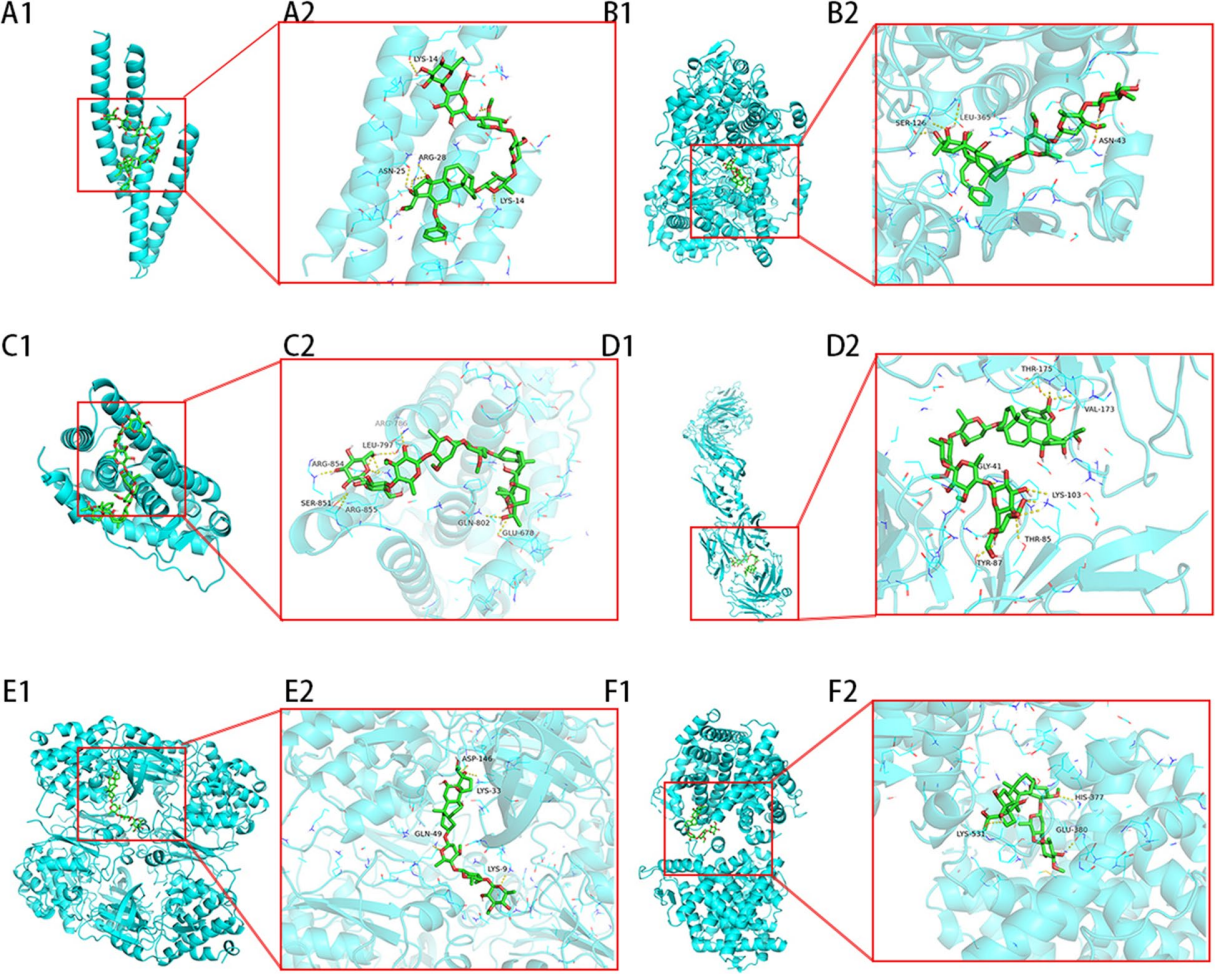
Molecular docking results of vital active components with target proteins. The blue portions represent the target proteins, while the green portions indicate the compounds. The yellow dashed lines represent the interactions between the ligands and the receptors. **A** JUN with Tenacissoside J, (**B**) PTGS2 with Marsdekoiside, (**C**) AR with Tenacissoside J, (**D**) VEGFA with Tenacissoside J, (**E**) CDK1 with Tenacissoside L, (**F**) ESR1 with Tenacissoside L form stable complex

To investigate the binding abilities of the active components in TGT with osteosarcoma targets, we performed molecular docking using the top ten target genes based on their degree values (EGFR, VEGFA, JUN, STAT3, HSP90AA1, ESR1, PTGS2, AR, HDAC1, and CDK1) as protein receptors, and the active components (Dihydroconduritol, D-oleandrose, Marsdekoiside, Tenacigenin B, Tenacissoside G, D-glucose, Saccharose, Taraxasterol, Tenacissoside J, and Tenacissoside L) as ligands. Figure 7 displays the binding energies of the compounds to the target proteins, where lower binding energy indicates better binding efficacy and more stable ligand-receptor interactions. Table 5 presents the spatial coordinates of each target and detailed information about the compound with the best binding efficacy. PyMOL 2.5.4 was used to visualize the compound-target interactions with the highest free binding energy score and their binding modes. Figure 8 illustrates the stable connections between the core components of TGT and the targets. Among them, CDK1, with the highest free binding energy, forms a stable complex with Tenacissoside L through residues ASP-146, LYS-33, GLN-49, and LYS-9.

**Table 5:** The binding energy information of the top ten target genes and their most stable binding compounds

Targets	PDB ID	UniProt ID	Center Coordinates	Compounds Name	Ingredient ID	Affinity (kcal/mol)
CDK1	6GU7	P06493	18.406,39.196,20.883	Tenacissoside L	HBIN045886	-10.7
PTGS2	5IKR	P35354	33.269,23.356,70.092	Marsdekoiside	HBIN034512	-10.3
VEGFA	1BJ1	P15692	21.648,-28.606,2.002	Tenacissoside J	HBIN045873	-8.9
STAT3	6NUQ	P40763	-2.228,19.134,24.624	Taraxasterol	HBIN045530	-8.5
EGFR	4WD5	P00533	-3.47,37.002,16.281	Taraxasterol	HBIN045530	-8.4
HSP90AA1	7UR3	P07900	-1.455,-14.041,0.109	Taraxasterol	HBIN045530	-8.4
JUN	5FV8	P05412	31.631,7.991,10.901	Tenacissoside J	HBIN045873	-8.1
ESR1	1A52	P03372	15.306,26.234,-45.16	Tenacissoside L	HBIN045886	-8.1
AR	5CJ6	P10275	6.786,27.71,11.101	Tenacissoside J	HBIN045873	-8.0
HDAC1	6Z2J	Q13547	215.989,215.989,222.46	Taraxasterol	HBIN045530	-7.9

### Pharmacokinetic evaluation of hub compounds

We conducted pharmacokinetic evaluations on compounds (Tenacigenin B, Marsdekoiside, Taraxasterol, Tenacissoside G, Tenacissoside L, and Tenacissoside J) with ligand binding energies lower than -8.0 kcal/mol using the SwissADME database. These compounds have an average molecular weight of 773.6 g/mol, are water-soluble, and exhibit moderate gastrointestinal absorption but are not likely to penetrate the blood–brain barrier (Table 6 and Table 7). All compounds, except Taraxasterol, are P-gp substrates. They did not show inhibitory activity against hepatic enzymes CYP1A2, CYP2C19, CYP2C9, CYP2D6, and CYP3A4 (Table 7). Tenacigenin B is highly absorbed in the gastrointestinal tract (Table 7). According to the predictions based on Lipinski and Veber's rules, Tenacigenin B and Taraxasterol exhibit good drug-likeness (Table 8).

**Table 6:** Physical and chemical properties of active ingredients in TGT

	Physicochemical Properties		
	Molecular weight(g/mol)	Solubility class	Consensus Log Po/w
Tenacigenin B	364.48 g/mol	Soluble	1.72
Marsdekoiside	963.15 g/mol	Soluble	2.98
Taraxasterol	426.72 g/mol	Poorly soluble	7.14
Tenacissoside G	792.95 g/mol	Soluble	3.4
Tenacissoside L	833.01 g/mol	Soluble	1.15
Tenacissoside J	1261.40 g/mol	Soluble	-0.58

**Table 7:** Pharmacokinetic characteristics of active ingredients in TGT

	Pharmacokinetics							
	GI absorption	BBB permeant	P-gp substrate	CYP1A2 inhibitor	CYP2C19 inhibitor	CYP2C9 inhibitor	CYP2D6 inhibitor	CYP3A4 inhibitor
Tenacigenin B	High	No	Yes	No	No	No	No	No
Marsdekoiside	Low	No	Yes	No	No	No	No	No
Taraxasterol	Low	No	No	No	No	No	No	No
Tenacissoside G	Low	No	Yes	No	No	No	No	No
Tenacissoside L	Low	No	Yes	No	No	No	No	No
Tenacissoside J	Low	No	Yes	No	No	No	No	No

**Table 8:** Drug-likeness of active ingredients in TGT

	Drug-likeness		
	Lipinski	Veber	Bioavailability Score
Tenacigenin B	Yes	Yes	0.55
Marsdekoiside	No	No	0.17
Taraxasterol	Yes	Yes	0.55
Tenacissoside G	No	No	0.17
Tenacissoside L	No	No	0.17
Tenacissoside J	No	No	0.17

### TGT inhibited osteosarcoma cell proliferation in vitro

**Fig. 9:**
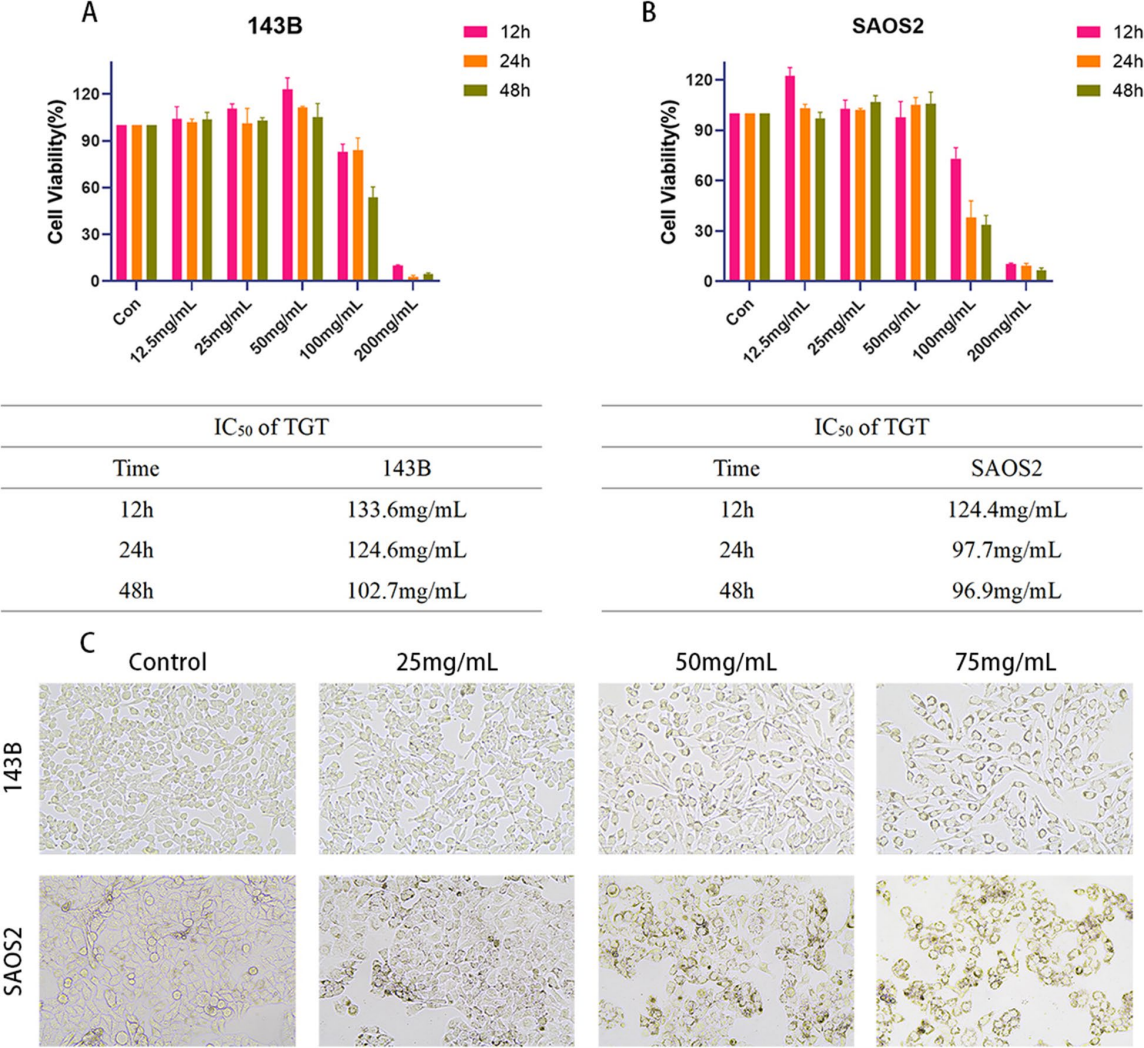
Inhibition of osteosarcoma cell proliferation by TGT. **A** Cell viability assay of 143B cells treated with different concentrations of TGT at 12, 24, and 48 h. **B** Cell viability assay of SAOS2 cells treated with varying concentrations of TGT at 12, 24, and 48 h. **C** Morphological changes in osteosarcoma cells (143B and SAOS2) treated with varying concentrations of TGT. Data are presented as mean ± SD (*n* = 5)

To validate the predicted anti-osteosarcoma effects of TGT from network pharmacology, we conducted CCK-8 assays to investigate its impact on osteosarcoma cell proliferation. Different concentrations of TGT (0, 12.5, 25, 50, 100, and 200 mg/mL) were applied to 143B and SAOS2 cells for viability assessment at 12, 24, and 48 h. The CCK-8 results showed that the IC_50_ values of TGT were 133.6 mg/mL, 124.6 mg/mL, and 102.7 mg/mL for 143B cells at 12, 24, and 48 h, respectively, and 124.4 mg/mL, 97.7 mg/mL, and 96.9 mg/mL for SAOS2 cells (Fig. 9A and B). These results indicate that TGT exhibits potent anti-osteosarcoma effects at high concentrations. To further investigate the morphological changes induced by TGT in osteosarcoma cells undergoing apoptosis, we observed the cell morphology under an inverted microscope after 48 h of treatment. As shown in Fig. 9C, apoptotic morphological changes were observed in osteosarcoma cells exposed to TGT. The control group exhibited a good growth state with irregular elliptical or spindle-shaped cells, while with increasing doses of the drug, cell density decreased, the arrangement became disordered, adhesion to the wall weakened, and cells gradually shrank and became round with cytoplasmic vacuolization.

### TGT-induced osteosarcoma cell apoptosis

**Fig. 10:**
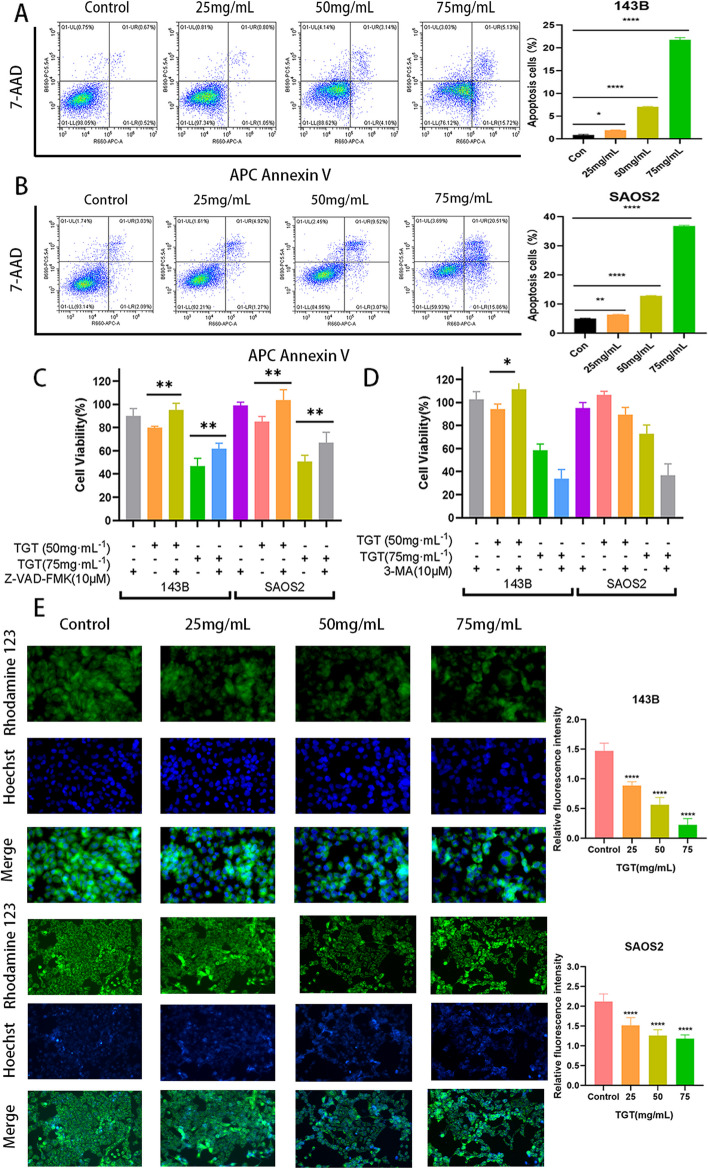
TGT induces apoptosis in osteosarcoma cells. (**A**-**B**) Flow cytometry detected that TGT induces apoptosis in 143B and SAOS2 cells. (**C**) The cytotoxic effect of TGT on osteosarcoma cells can be reversed by co-administration with the apoptosis inhibitor Z-VAD-FMK. (**D**) Co-administration of TGT with the autophagy inhibitor 3-MA only reverses the cytotoxic effect on osteosarcoma 143B cells at low concentrations. (**E**) Changes in MMP in osteosarcoma cells treated with TGT observed under fluorescence microscopy. Data are presented as mean ± SD (*n* = 5). *P < 0.1, **P < 0.01, and ****P < 0.0001 versus the Control group

To determine the mode of cell death involved in the reduction of osteosarcoma cell viability by TGT, we treated 143B and SAOS2 cells with TGT at concentrations of 0, 25, 50, and 75 mg/mL, followed by staining using APC Annexin V in combination with 7-AAD. The results of APC Annexin V/7-AAD double staining showed that TGT induced apoptosis in a dose-dependent manner in 143B and SAOS2 cells at concentrations of 25, 50, and 75 mg/mL (Fig. 10A and B). When TGT (at 50 and 75 mg/mL) was co-administered with the pan-Caspase inhibitor Z-VAD-FMK (10 μM), the inhibitory effect of TGT on the proliferation of 143B and SAOS2 cells was reversed (Fig. 10C). This indicates that the induction of apoptosis in osteosarcoma cells by TGT is caspase-dependent. On the other hand, when TGT was co-administered with the autophagy inhibitor 3-MA (10 μM), it only reversed the cytotoxic effect of TGT on 143B cells at low concentrations. In contrast, the cytotoxic effect on SAOS2 cells was more significant (Fig. 10D). This suggests that the induction of autophagy is not the leading cause of cell death. Therefore, these data indicate that the induction of cell death in osteosarcoma cells by TGT is mainly mediated by caspase-dependent apoptosis.

### TGT-induced mitochondrial apoptosis in osteosarcoma cells

During cellular apoptosis, the loss of mitochondrial transmembrane potential (ΔΨm) leads to the efflux of rhodamine 123 from the mitochondria, resulting in reduced fluorescence. Based on the observations made using fluorescence microscopy, it was found that compared to the control group, the TGT-treated group exhibited a dose-dependent decrease in MMP (Fig. 10E). This indicates that TGT can induce tumor cell apoptosis by disrupting the stability of the mitochondrial membrane.

### TGT modulated apoptosis-related protein activity in osteosarcoma cells

**Fig. 11:**
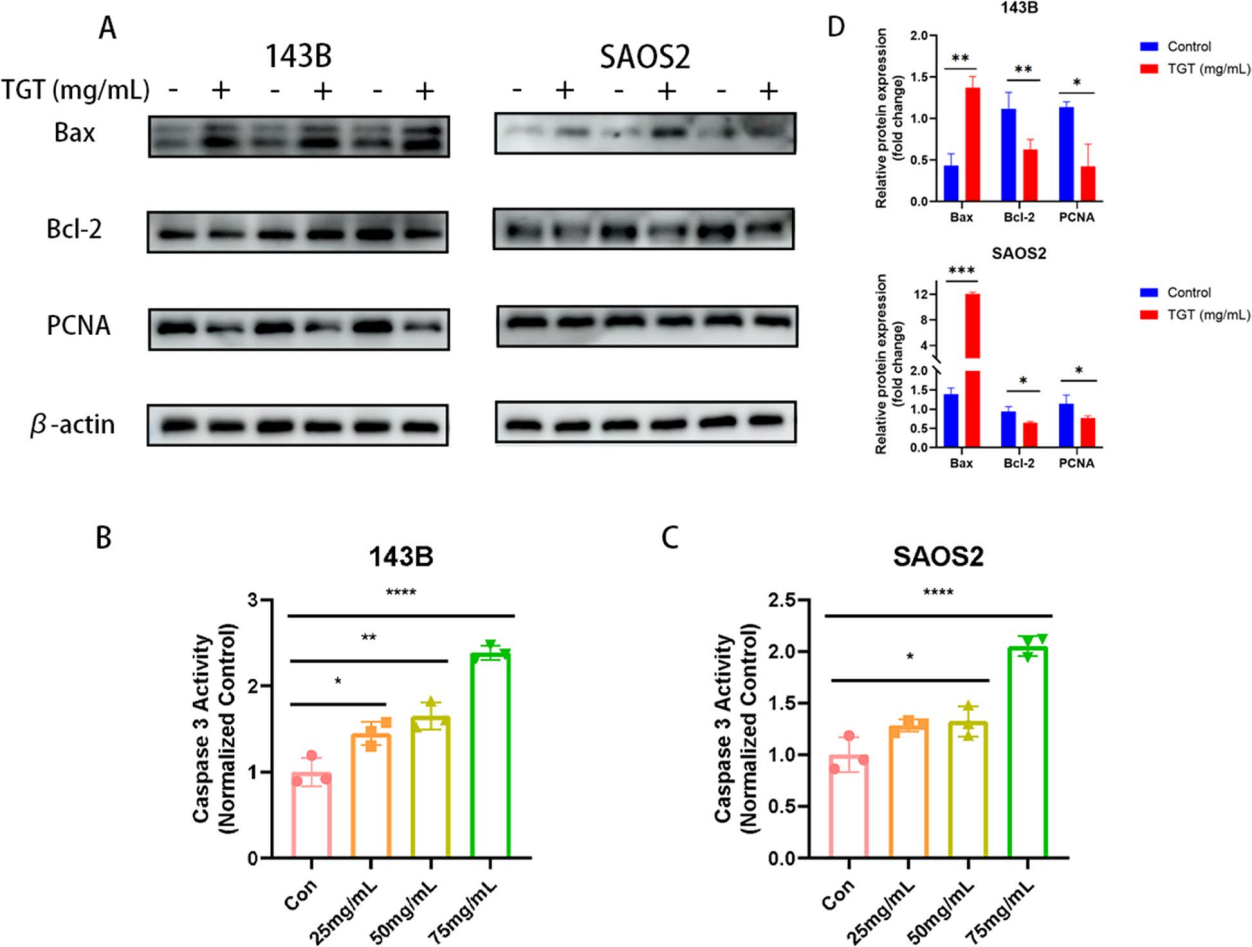
Modulation of apoptosis-related protein expression in osteosarcoma cells by TGT. **A** TGT significantly promotes the expression of the pro-apoptotic protein Bax and inhibits the expression of the anti-apoptotic protein Bcl-2 and the proliferation-related protein PCNA. The negative sign (-) represents the control group, while the plus sign ( +) represents the group treated with TGT at a 75 mg/mL concentration. Moreover, TGT significantly enhances the Caspase-3 enzyme activity in (**B**) 143B cells and (**C**) SAOS2 cells. Data are presented as mean ± SD (*n* = 3). *P < 0.1, **P < 0.01, and ****P < 0.0001 versus the Control group

The expression of apoptosis-related proteins was analyzed through Western blotting. The Caspase-3 enzyme activity in 143B and SAOS2 cells was measured using an enzyme-linked immunosorbent assay (ELISA). The pro-apoptotic protein Bax and anti-apoptotic protein Bcl-2 can reflect the cellular apoptotic status, while Caspase-3 is a key enzyme involved in cell apoptosis. PCNA can indicate cell proliferation status and vitality. As shown in Fig. 11, TGT significantly promoted the expression of Bax and suppressed the expression of Bcl-2 and PCNA while it increased Caspase-3 enzyme activity. Therefore, TGT can induce apoptosis in 143B and SAOS2 cells by upregulating pro-apoptotic proteins and inhibiting the expression of anti-apoptotic proteins.

### TGT regulated the expression of critical genes in osteosarcoma

**Fig. 12:**
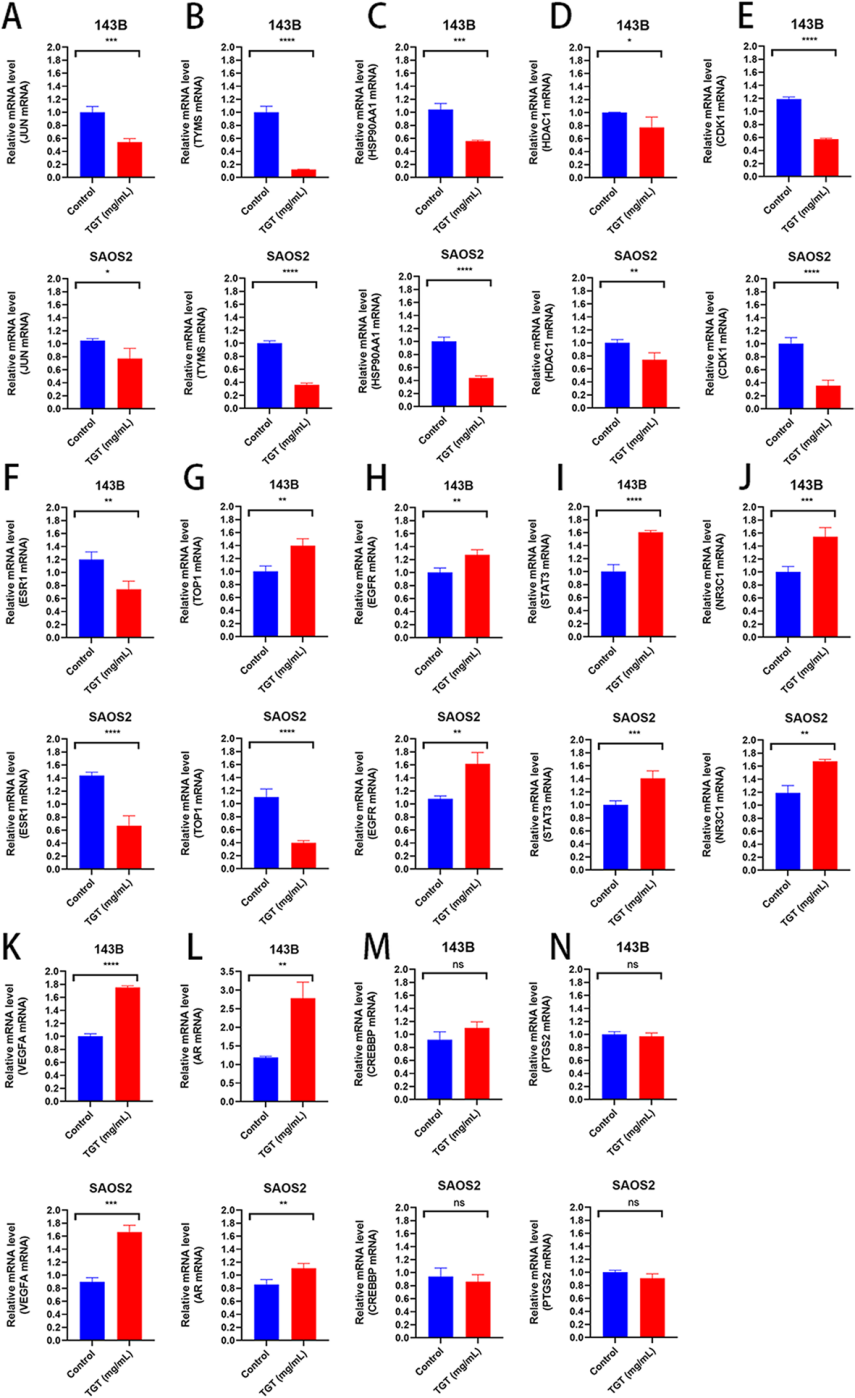
TGT regulates the expression of key target genes in osteosarcoma cells 143B and SAOS2. The concentration of TGT is 75 mg/mL. **A** JUN, (**B**) TYMS, (**C**) HSP90AA1, (**D**) HDAC1, (**E**) CDK1, (**F**) ESR1, (**G**) TOP1, (**H**) EGFR, (**I**) STAT3, (**J**) NR3C1, (**K**) VEGFA, (**L**) AR, (**M**) CREBBP, and (**N**) PTGS2. Data are presented as mean ± SD (*n* = 4).*P < 0.1, **P < 0.01, ***P < 0.001 and ****P < 0.0001 versus the Control group

To investigate the molecular mechanism of TGT against osteosarcoma, we compared the differential expression of 14 essential genes between the control group and the drug-treated group using RT-PCR experiments (Fig. 12). Compared to the control group after 48 h of treatment with TGT, the expression of genes JUN, TYMS, HSP90AA1, HDAC1, CDK1, and ESR1 was downregulated, which showed inhibitory effect on tumor cell proliferation. On the other hand, the expression of genes TOP1, EGFR, STAT3, NR3C1, VEGFA, and AR was upregulated, which could promote tumor cell growth. No significant differences were observed in the expression of CREBBP and PTGS2. These results suggest that JUN, TYMS, HSP90AA1, HDAC1, CDK1, and ESR1 may serve as core drug targets for treating osteosarcoma with TGT.

## Discussion

Although surgery combined with chemotherapy has dramatically improved the prognosis of patients with osteosarcoma, the five-year survival rate of patients with osteosarcoma has not been significantly improved in recent decades due to the intolerance of traditional platinum-based chemotherapy drugs [20, 21]. Hence, there is a pressing requirement to explore novel potent medications and/or approaches to address osteosarcoma. Over the past few years, numerous research studies have suggested that TCM has the potential to mitigate the adverse reactions of chemotherapy, boost the immune system, and facilitate the process of apoptosis and elimination of cancerous cells [22–24]. These studies have gained increasing attention from people. TCM preparation TGT exhibits significant therapeutic effects on various tumors, such as triggering cell cycle halt and apoptosis in ovarian cancer [19, 25]. Additionally, it synergizes with chemotherapy drugs to enhance the efficacy against gastric cancer while reducing adverse reactions [10]. Moreover, it induces immunogenic cell death associated with endoplasmic reticulum stress in non-small cell lung cancer cells and causes cell cycle arrest in esophageal cancer cells [9, 26]. Nonetheless, the therapeutic process of TGT for osteosarcoma is still not understood. TGT is a TCM formulation that typically contains multiple components to interact with various targets, making it complicated for traditional research methods. Hence, this investigation employs network pharmacology in conjunction with molecular biology techniques to reveal the cytotoxic impacts and molecular pathways of TGT in osteosarcoma.

Based on the findings from network pharmacology and molecular docking, Tenacigenin B, Marsdekoiside, Taraxasterol, Tenacissoside G, Tenacissoside L, and Tenacissoside J were identified as the primary bioactive constituents in TGT. It has been demonstrated in prior research that the antitumor effectiveness of paclitaxel in nude mice can be improved by Tenacigenin B through the inhibition of CYP3A4, which is an enzyme responsible for drug metabolism [27]. The induction of cell cycle arrest and upregulation of apoptosis-related proteins Bax and caspase-9 by Taraxasterol can facilitate apoptosis in non-small cell lung cancer [28]. The extent of DNA damage and phosphorylation of p53 can be increased by Tenacissoside G, thereby enhancing the anti-colorectal cancer effects of 5-FU [29]. Nevertheless, the scarcity of literature regarding Marsdekoiside, Tenacissoside L, and Tenacissoside J suggests additional experimental investigation into these crucial substances. These data indicate that TGT may exert clinical efficacy through the synergistic actions of multiple active ingredients.

Considering the pharmaceutical value of active monomers, we used the SwissADME database to predict the compounds' physicochemical properties, pharmacokinetic characteristics, and drug-likeness. Our study found that Tenacigenin B and Taraxasterol exhibited favorable drug-like properties. They are water-soluble and cannot cross the blood–brain barrier, thus reducing the risk of damage to the central nervous system. Moreover, they did not show inhibitory effects on liver enzymes, which suggests that they would not interfere with the metabolism and clearance of other drugs in the body, thereby minimizing the risk of drug-drug interactions.

Through network analysis, we identified fourteen key targets: JUN, TYMS, HSP90AA1, HDAC1, CDK1, ESR1, TOP1, EGFR, STAT3, NR3C1, VEGFA, AR, CREBBP, and PTGS2. Research has shown that inhibiting JUN activity can reduce the invasiveness of osteosarcoma cells [30]. HSP90AA1 promotes drug resistance in osteosarcoma cells, such as resistance to chemotherapy drugs like doxorubicin, cisplatin, and methotrexate, by inducing autophagy [31]. HDAC1 can promote the proliferation and invasion of osteosarcoma cells by inhibiting the transcription of the MicroRNA-326 gene [32]. After the suppression of CDK1 expression, it can induce cell cycle arrest at the G2/M phase, thereby inhibiting the proliferation of osteosarcoma cells [33]. Overall, network pharmacology analysis indicates that TGT exhibits a robust inhibitory effect on osteosarcoma cell proliferation, providing potential therapeutic strategies for osteosarcoma treatment. Consistent with the network pharmacology results, the RT-PCR experiments showed significant downregulation of JUN, HSP90AA1, HDAC1, and CDK1 expression by TGT, highlighting their potential significance as targets in the anti-osteosarcoma mechanism of TGT. Additionally, co-administration of TGT with EGFR inhibitors, STAT3 inhibitors, and VEGFA inhibitors may enhance the anti-osteosarcoma effects of TGT, further inhibiting the development and metastasis of osteosarcoma.

Target organ analysis revealed that the targets of TGT are mainly distributed in the body's immune system and are present in various tissues and organs throughout the body. Existing literature has reported that TGT can enhance the safety and efficacy of colorectal cancer treatment [34]. At the same time, TGT can improve the effectiveness of chemotherapy drugs by regulating the immune function of the human body, thereby improving the prognosis of advanced gastric cancer patients [10]. This suggests that TGT not only exerts its effects on local bone tissue but also has the potential to impact systemic immune regulatory responses, providing protective effects on various organs within the body.

Apoptosis, as a programmed cell death mechanism, has gained significant attention in the pathogenesis of osteosarcoma [35]. Induction of apoptosis can effectively inhibit the growth and metastasis of osteosarcoma, making the induction of tumor cell apoptosis by TCM a potentially effective approach for treating osteosarcoma [36]. In this study, GO functional analysis indicates that TGT plays a role in inhibiting osteosarcoma through involvement in various biological processes, such as negative regulation of transcription from RNA polymerase II promoter and positive regulation of peptidyl-serine phosphorylation. By modulating the transcription activity of cancer genes and tumor suppressor genes through RNA polymerase II promoter and peptidyl-serine phosphorylation, it can inhibit the proliferation of cancer cells, induce tumor cell apoptosis, and ultimately improve the prognosis of tumor patients [37, 38]. The CC enrichment analysis of TGT indicates that it is enriched in the cytoplasm, nucleus, nucleoplasm, and mitochondrial matrix. This suggests that TGT may induce mitochondrial apoptosis in osteosarcoma cells. Mitochondria, as crucial organelles, can regulate cellular energy production, calcium homeostasis, and redox balance. When damaged, mitochondria can trigger mitochondrial-mediated cell apoptosis [39]. Consistent with the results of network pharmacology, biological experiments have demonstrated that TGT can induce morphological changes and mitochondrial apoptosis in osteosarcoma cells. KEGG enrichment analysis revealed that the HIF-1 signaling pathway and PD-L1 expression and PD-1 checkpoint pathway in cancer may be the main signaling pathways in the network. The HIF-1 signaling pathway regulates inflammation, angiogenesis, and proliferation/apoptosis balance and promotes tumor invasion and metastasis under hypoxic and anaerobic conditions [40, 41]. The immune checkpoint PD-1 on T cell surfaces, when bound to its ligand PD-L1 on tumor cells, can inhibit the immune-mediated killing of tumor cells and facilitate immune evasion by tumor cells [42]. Previous studies have indicated that inhibiting the HIF-1 signaling pathway and targeting the suppression of PD-L1 expression may induce apoptosis in osteosarcoma cells [43, 44]. The research shows that inhibiting C-JUN expression can suppress the expression and secretion of PD-L1 [45]. In addition, the inhibition of HSP90AA1 expression can increase the sensitivity of immune-refractory tumors to cellular immunotherapy and PD-1 blockade [46]. RT-PCR experiments also demonstrate that TGT can promote anti-tumor immune response by downregulating the expression of C-JUN and HSP90AA1. These findings suggest that TGT may exert its anti-osteosarcoma effects by inhibiting tumor cell growth and metastasis and inducing immune responses through immune checkpoint blockade. At the same time, the HIF-1 signaling pathway and PD-L1 expression and PD-1 checkpoint pathway in cancer are the main pathways in the network. They may be associated with TGT-induced apoptosis in osteosarcoma.

To test this hypothesis further, we performed in vitro pharmacological experiments using TGT in osteosarcoma cells. According to the results of the CCK-8 assay, a high dose of TGT had the best cytotoxic effect on osteosarcoma cells within 48 h, significantly reducing their survival rate. Subsequent experiments utilized doses below the IC_50_ to mitigate the possible cytotoxic effects on normal cells. Morphological experiments showed that after TGT stimulation, the number of tumor cells decreased and the cell morphology was irregularly destroyed. Flow cytometry instrument and MMP detection results show that the TGT can induce osteosarcoma cells induced apoptosis dose-dependently. In addition, western blotting assay and Caspase-3 activity assay further confirmed that TGT caused the expression of apoptotic proteins Caspase-3 and Bax while down-regulating the expression of Bcl-2 and PCNA. In conclusion, our results show that the TGT may be a potential strategy for treating osteosarcoma.

## Conclusions

This study aims to systematically investigate the targets and pathways of TGT in treating osteosarcoma using network pharmacology and molecular docking techniques. These bioinformatics methods indicate that Tenacigenin B, Marsdekoiside, Taraxasterol, Tenacissoside G, Tenacissoside L, and Tenacissoside J are the primary active components of the TGT. By suppressing the activities of JUN, HSP90AA1, HDAC1, and CDK1, TGT can impede the growth of osteosarcoma cells. Moreover, it might stimulate the immune system, leading to an immune-regulatory reaction within the body. Moreover, TGT triggers cell apoptosis in osteosarcoma cells by controlling the levels of Bax and Bcl-2. The findings of our study offer a fresh theoretical foundation and technical assistance for utilizing TGT as a supplementary medication in the management of osteosarcoma.

### Supplementary Information

**Additional file 1: Supplementary Table 1.** Primer sequences for the genes.

**Additional file 2: Supplementary Table 2.** Distribution of target genes of TGT in organs.

**Additional file 3. **

## Data Availability

The data for the current study were obtained from Herb database (http://herb.ac.cn/), PubChem (https://pubchem.ncbi.nlm.nih.gov/) database, SwissTargetPrediction (https://www.swisstargetprediction.ch/) database, Uniprot (https://www.uniprot.org/) database, GEO (www.ncbi.nlm.nih.gov/geo/) database, GeneCards (http://www.genecards.org/) database, DisGeNET (https://www.disgenet.org/) database, DrugBank (https://go.drugbank.com/) database, STRING (https://string-db.org/) database, BioGPS (https://biogps.org) database, DAVID (https://david.ncifcrf.gov) database, PDB (https://www.rcsb.org/) database and SwissADME (http://www.swissadme.ch/) database.

## Abbreviations

TGT: Tongguanteng injection
PPI: Protein-protein interaction
MMP: Mitochondrial membrane potential
TCM: Traditional chinese medicine
DEGs: Differentially expressed genes
BC: Betweenness centrality
CC: Closeness centrality
DC: Degree centrality
GO: Gene ontology
KEGG: Kyoto encyclopedia of genes and genomes
DMEM: Dulbecco's modified eagle's medium
FBS: Fetal bovine serum
CCK-8: Cell counting kit-8
3-MA: 3-Methyladenine
RT-PCR: Real-time polymerase chain reaction
ANOVA: One-way analysis of variance
ELISA: Enzyme-linked immunosorbent assay
