# Supplementary material for: Network pharmacology and experimental validation to study the potential mechanism of Tongguanteng injection in regulating apoptosis in osteosarcoma
Source: BMC Complement Med Ther. 2024 Jan 31;24:67. doi: 10.1186/s12906-024-04354-z (PMC10829404; doi:10.1186/s12906-024-04354-z)
Supplement: Supplementary file 1 — Additional file 1: Supplementary Table 1. Primer sequences for the genes. [file 12906_2024_4354_MOESM1_ESM.doc]

**Supplementary table 1** Primer sequences for the genes

| Primer | Forward primer | Reverse primer |  |
| --- | --- | --- | --- |
| EGFR | ACAGCATAGACGACACCTTCCTC | TGGCTTGGACACTGGAGACTG |  |
| VEGFA | GGAGGAGGAAGAAGAGAAGGAAGAG | GCGGCTGGAGCACTGTCTG |  |
| JUN | GCCAAGAACTCGGACCTCCTC | TCGGCGTGGTGGTGATGTG |  |
| STAT3 | ACCAAGCGAGGACTGAGCATC | CAGCCAGACCCAGAAGGAGAAG |  |
| HSP90AA1 | ATCCACCACTCTACTCTGTCTCTG | CTCAACCTCCTCCTCCTCCATC |  |
| ESR1 | TCTGAGGCTGCGGCGTTC | GGCTCGTTCTCCAGGTAGTAGG |  |
| PTGS2 | GGTGATGAGCAGTTGTTCCAGAC | GGGTGTTAAATTCAGCAGCAATACG |  |
| AR | GCAGCAGCAGCAGCAAGAG | CTCATCCAGGACCAGGTAGCC |  |
| HDAC1 | GCTCCACATCAGTCCTTCCAATATG | CCTCATCGCCACTCTCCTCAG |  |
| CDK1 | TCAGTCTTCAGGATGTGCTTATGC | TGTACTGACCAGGAGGGATAGAATC |  |
| NR3C1 | TCAGACTCCAAGCAGCGAAGAC | GAATCCCAGGTCATTTCCCATCAC |  |
| CREBBP | TGCCTGGTGCTGCTCTTCC | TGGTGTCGTGTGCTGGAGAG |  |
| TOP1 | CAAGGTTCCTTCTCCTCCTCCAG | TTCAGCCGCCGAGCAGTC |  |
| TYMS | AGCGAGAACCCAGACCTTTCC | AAGCACCCTAAACAGCCATTTCC |  |
