# Supplementary material for: Network pharmacology and experimental validation to study the potential mechanism of Tongguanteng injection in regulating apoptosis in osteosarcoma
Source: BMC Complement Med Ther. 2024 Jan 31;24:67. doi: 10.1186/s12906-024-04354-z (PMC10829404; doi:10.1186/s12906-024-04354-z)
Supplement: Supplementary file 2 — Additional file 2: Supplementary Table 2. Distribution of target genes of TGT in organs. [file 12906_2024_4354_MOESM2_ESM.doc]

**Supplementary table 2** Distribution of target genes of TGT in organs

| Term | Gene |
| --- | --- |
| 721_B_lymphoblasts | AURKB |
| 721_B_lymphoblasts | CDK1 |
| 721_B_lymphoblasts | DTYMK |
| 721_B_lymphoblasts | EIF4E |
| 721_B_lymphoblasts | HDAC1 |
| 721_B_lymphoblasts | HMGCR |
| 721_B_lymphoblasts | NR3C1 |
| 721_B_lymphoblasts | POLA1 |
| 721_B_lymphoblasts | POLD1 |
| 721_B_lymphoblasts | RAF1 |
| 721_B_lymphoblasts | TOP1 |
| 721_B_lymphoblasts | TYMS |
| Adipocyte | AR |
| Adipocyte | HMGCR |
| Adipocyte | HSD11B1 |
| Adipocyte | LGALS3 |
| Adipocyte | MMP2 |
| Adipocyte | NR1H3 |
| Adipocyte | PFKFB3 |
| Adipocyte | STAT3 |
| AdrenalCortex | ABCB1 |
| AdrenalCortex | HMGCR |
| Adrenalgland | ABCB1 |
| Adrenalgland | MMP2 |
| Amygdala | HMGCR |
| Amygdala | HSP90AA1 |
| Amygdala | PFKFB3 |
| Appendix | LGALS3 |
| Appendix | MMP2 |
| BDCA4+_DentriticCells | HDAC1 |
| BDCA4+_DentriticCells | JUN |
| BDCA4+_DentriticCells | KDM2A |
| BDCA4+_DentriticCells | NR3C1 |
| BDCA4+_DentriticCells | PTGS2 |
| BDCA4+_DentriticCells | RAF1 |
| BDCA4+_DentriticCells | TOP1 |
| BDCA4+_DentriticCells | TYMS |
| Bonemarrow | AURKB |
| Bonemarrow | CXCR2 |
| Bonemarrow | MMP8 |
| Bonemarrow | TYMS |
| BronchialEpithelialCells | HDAC1 |
| BronchialEpithelialCells | HMGCR |
| BronchialEpithelialCells | HSD11B1 |
| BronchialEpithelialCells | LGALS3 |
| BronchialEpithelialCells | PTGS2 |
| BronchialEpithelialCells | TOP1 |
| BronchialEpithelialCells | TYMS |
| CardiacMyocytes | AURKB |
| CardiacMyocytes | CDK1 |
| CardiacMyocytes | DPP4 |
| CardiacMyocytes | MMP2 |
| CardiacMyocytes | TYMS |
| CD105+_Endothelial | AURKB |
| CD105+_Endothelial | CDK1 |
| CD105+_Endothelial | DTYMK |
| CD105+_Endothelial | EIF4E |
| CD105+_Endothelial | HDAC1 |
| CD105+_Endothelial | LGALS3 |
| CD105+_Endothelial | POLA1 |
| CD105+_Endothelial | POLD1 |
| CD105+_Endothelial | TOP1 |
| CD105+_Endothelial | TYMS |
| CD14+_Monocytes | CXCR2 |
| CD14+_Monocytes | HDAC1 |
| CD14+_Monocytes | HMGCR |
| CD14+_Monocytes | JUN |
| CD14+_Monocytes | LGALS3 |
| CD14+_Monocytes | NR3C1 |
| CD14+_Monocytes | PTGS2 |
| CD14+_Monocytes | RAF1 |
| CD14+_Monocytes | TOP1 |
| CD19+_BCells(neg | HDAC1 |
| CD19+_BCells(neg | JUN |
| CD19+_BCells(neg | KDM2A |
| CD19+_BCells(neg | NR3C1 |
| CD19+_BCells(neg | RAF1 |
| CD33+_Myeloid | CDK1 |
| CD33+_Myeloid | HDAC1 |
| CD33+_Myeloid | HMGCR |
| CD33+_Myeloid | JUN |
| CD33+_Myeloid | KDM2A |
| CD33+_Myeloid | LGALS3 |
| CD33+_Myeloid | NR3C1 |
| CD33+_Myeloid | PFKFB3 |
| CD33+_Myeloid | PTGS2 |
| CD33+_Myeloid | RAF1 |
| CD33+_Myeloid | STAT3 |
| CD33+_Myeloid | TOP1 |
| CD33+_Myeloid | TYMS |
| CD34+ | AURKB |
| CD34+ | CDK1 |
| CD34+ | DTYMK |
| CD34+ | EIF4E |
| CD34+ | HDAC1 |
| CD34+ | NR3C1 |
| CD34+ | POLA1 |
| CD34+ | POLD1 |
| CD34+ | RAF1 |
| CD34+ | TOP1 |
| CD34+ | TYMS |
| CD4+_Tcells | ABCB1 |
| CD4+_Tcells | HDAC1 |
| CD4+_Tcells | KDM2A |
| CD4+_Tcells | NR3C1 |
| CD4+_Tcells | RAF1 |
| CD56+_NKCells | ABCB1 |
| CD56+_NKCells | CXCR2 |
| CD56+_NKCells | EIF4E |
| CD56+_NKCells | HDAC1 |
| CD56+_NKCells | KDM2A |
| CD56+_NKCells | NR3C1 |
| CD56+_NKCells | RAF1 |
| CD56+_NKCells | STAT3 |
| CD56+_NKCells | TYMS |
| CD71+_EarlyErythroid | AURKB |
| CD71+_EarlyErythroid | CDK1 |
| CD71+_EarlyErythroid | HDAC1 |
| CD71+_EarlyErythroid | LGALS3 |
| CD71+_EarlyErythroid | POLA1 |
| CD71+_EarlyErythroid | TOP1 |
| CD71+_EarlyErythroid | TYMS |
| CD8+_Tcells | ABCB1 |
| CD8+_Tcells | HDAC1 |
| CD8+_Tcells | KDM2A |
| CD8+_Tcells | NR3C1 |
| CD8+_Tcells | RAF1 |
| Colon | EIF4E |
| Colon | HDAC1 |
| Colon | HMGCR |
| Colon | HSD11B2 |
| Colon | LGALS3 |
| Colon | MMP2 |
| Colon | TYMS |
| Colon | VDR |
| Colorectaladenocarcinoma | AURKB |
| Colorectaladenocarcinoma | POLD1 |
| Colorectaladenocarcinoma | TYMS |
| Fetalbrain | HMGCR |
| Fetalbrain | PFKFB3 |
| Fetalliver | CYP19A1 |
| Fetalliver | HMGCR |
| Fetalliver | TOP1 |
| Fetalliver | TYMS |
| Fetallung | HSD11B1 |
| Fetallung | JUN |
| Fetallung | KDR |
| Fetallung | MMP2 |
| Fetallung | PTGS2 |
| Fetallung | TOP1 |
| Fetallung | TYMS |
| FetalThyroid | MMP2 |
| FetalThyroid | RAF1 |
| Heart | IL2 |
| Heart | LGALS3 |
| Heart | MMP2 |
| Hypothalamus | FGF1 |
| Hypothalamus | PFKFB3 |
| Hypothalamus | TYMS |
| Kidney | DPP4 |
| Kidney | HSD11B2 |
| Leukemia_chronicMyelogenousK-562 | AURKB |
| Leukemia_chronicMyelogenousK-562 | CDK1 |
| Leukemia_chronicMyelogenousK-562 | HMGCR |
| Leukemia_chronicMyelogenousK-562 | TYMS |
| Leukemia_promyelocytic-HL-60 | AURKB |
| Leukemia_promyelocytic-HL-60 | CDK1 |
| Leukemia_promyelocytic-HL-60 | EIF4E |
| Leukemia_promyelocytic-HL-60 | HDAC1 |
| Leukemia_promyelocytic-HL-60 | HMGCR |
| Leukemia_promyelocytic-HL-60 | TOP1 |
| Leukemia_promyelocytic-HL-60 | TYMS |
| Leukemialymphoblastic(MOLT-4) | AURKB |
| Leukemialymphoblastic(MOLT-4) | CDK1 |
| Leukemialymphoblastic(MOLT-4) | HDAC1 |
| Leukemialymphoblastic(MOLT-4) | HMGCR |
| Leukemialymphoblastic(MOLT-4) | POLD1 |
| Leukemialymphoblastic(MOLT-4) | TOP1 |
| Leukemialymphoblastic(MOLT-4) | TYMS |
| Liver | AR |
| Liver | HSD11B1 |
| Liver | NR1H3 |
| Lung | JUN |
| Lung | LGALS3 |
| Lung | MMP2 |
| Lung | PFKFB3 |
| Lymphnode | KDM2A |
| Lymphnode | NR1H3 |
| Lymphoma_burkitts(Daudi) | AURKB |
| Lymphoma_burkitts(Daudi) | CDK1 |
| Lymphoma_burkitts(Daudi) | HDAC1 |
| Lymphoma_burkitts(Daudi) | POLD1 |
| Lymphoma_burkitts(Daudi) | TOP1 |
| Lymphoma_burkitts(Daudi) | TYMS |
| Lymphoma_burkitts(Raji) | HDAC1 |
| Lymphoma_burkitts(Raji) | POLD1 |
| Lymphoma_burkitts(Raji) | TOP1 |
| Lymphoma_burkitts(Raji) | TYMS |
| OlfactoryBulb | FGF1 |
| OlfactoryBulb | LGALS3 |
| OlfactoryBulb | MMP2 |
| OlfactoryBulb | NR1H3 |
| OlfactoryBulb | NR3C1 |
| OlfactoryBulb | PFKFB3 |
| Ovary | HSD11B1 |
| Ovary | MMP2 |
| Ovary | STAT3 |
| Pancreas | JUN |
| Pancreas | LGALS3 |
| Pancreas | VEGFA |
| PancreaticIslet | MMP2 |
| PancreaticIslet | NR3C1 |
| PancreaticIslet | PTGS2 |
| PancreaticIslet | STAT3 |
| PancreaticIslet | VEGFA |
| Pineal_day | EIF4E |
| Pineal_day | HSP90AA1 |
| Pineal_day | KDM2A |
| Pineal_day | RAF1 |
| Pineal_day | TOP1 |
| Pineal_night | EIF4E |
| Pineal_night | KDM2A |
| Pineal_night | KDR |
| Pineal_night | MMP2 |
| Pineal_night | RAF1 |
| Pineal_night | TOP1 |
| Pituitary | NR3C1 |
| Placenta | CYP19A1 |
| Placenta | EGFR |
| Placenta | HSD11B1 |
| Placenta | HSD11B2 |
| Placenta | KDR |
| Placenta | MMP2 |
| Placenta | TYMS |
| PrefrontalCortex | FGF1 |
| PrefrontalCortex | HSP90AA1 |
| PrefrontalCortex | KDM2A |
| PrefrontalCortex | NR3C1 |
| PrefrontalCortex | PFKFB3 |
| Prostate | DPP4 |
| Prostate | EGFR |
| Prostate | FOLH1 |
| Prostate | JUN |
| Prostate | LGALS3 |
| Prostate | MMP2 |
| Prostate | NR3C1 |
| Prostate | PTGS2 |
| Prostate | VEGFA |
| Retina | EIF4E |
| Retina | FGF1 |
| Retina | FOLH1 |
| Retina | JUN |
| Retina | KDM2A |
| Retina | KDR |
| Retina | MMP2 |
| Retina | PFKFB3 |
| Retina | RAF1 |
| Retina | STAT3 |
| Salivarygland | FOLH1 |
| Salivarygland | HSD11B2 |
| SkeletalMuscle | PFKFB3 |
| Skin | LGALS3 |
| Skin | MMP2 |
| Small_intestine | DPP4 |
| Small_intestine | EIF4E |
| Small_intestine | HDAC1 |
| Small_intestine | HSD11B2 |
| Small_intestine | KDR |
| Small_intestine | LGALS3 |
| Small_intestine | MMP2 |
| Small_intestine | TYMS |
| Small_intestine | VDR |
| SmoothMuscle | DPP4 |
| SmoothMuscle | FGF2 |
| SmoothMuscle | HSD11B1 |
| SmoothMuscle | MMP2 |
| SmoothMuscle | NR3C1 |
| SmoothMuscle | PTGS2 |
| SmoothMuscle | STAT3 |
| SmoothMuscle | TYMS |
| Spinalcord | FGF1 |
| Spinalcord | JUN |
| Spinalcord | PFKFB3 |
| SuperiorCervicalGanglion | CXCR2 |
| SuperiorCervicalGanglion | FOLH1 |
| SuperiorCervicalGanglion | PRKCA |
| Testis | MMP2 |
| Testis | NR1H3 |
| Testis | TYMS |
| TestisGermCell | HSD11B1 |
| TestisGermCell | LGALS3 |
| TestisGermCell | MMP2 |
| TestisGermCell | TYMS |
| TestisIntersitial | TYMS |
| TestisLeydigCell | MMP2 |
| TestisLeydigCell | TYMS |
| TestisSeminiferousTubule | TYMS |
| Thalamus | HSD11B1 |
| Thymus | AURKB |
| Thymus | HDAC1 |
| Thymus | KDM2A |
| Thymus | NR3C1 |
| Thymus | TYMS |
| Thyroid | EIF4E |
| Thyroid | FLT1 |
| Thyroid | HDAC1 |
| Thyroid | JUN |
| Thyroid | KDR |
| Thyroid | VEGFA |
| Tongue | LGALS3 |
| Tonsil | MMP12 |
| Tonsil | NR3C1 |
| Tonsil | TYMS |
| Trachea | JUN |
| Trachea | LGALS3 |
| Trachea | MMP2 |
| Uterus | JUN |
| Uterus | KDR |
| Uterus | LGALS3 |
| Uterus | MMP2 |
| Uterus | NR3C1 |
| UterusCorpus | LGALS3 |
| UterusCorpus | MMP2 |
| UterusCorpus | VEGFA |
| WholeBlood | CXCR1 |
| WholeBlood | CXCR2 |
| WholeBlood | HDAC1 |
| WholeBlood | KDM2A |
| WholeBlood | NR3C1 |
| WholeBlood | PTGS2 |
| WholeBlood | RAF1 |
| WholeBlood | STAT3 |
| WholeBlood | TOP1 |
