# Supplementary material for: Network pharmacology and experimental validation to study the potential mechanism of Tongguanteng injection in regulating apoptosis in osteosarcoma
Source: BMC Complement Med Ther. 2024 Jan 31;24:67. doi: 10.1186/s12906-024-04354-z (PMC10829404; doi:10.1186/s12906-024-04354-z)

143B BAX

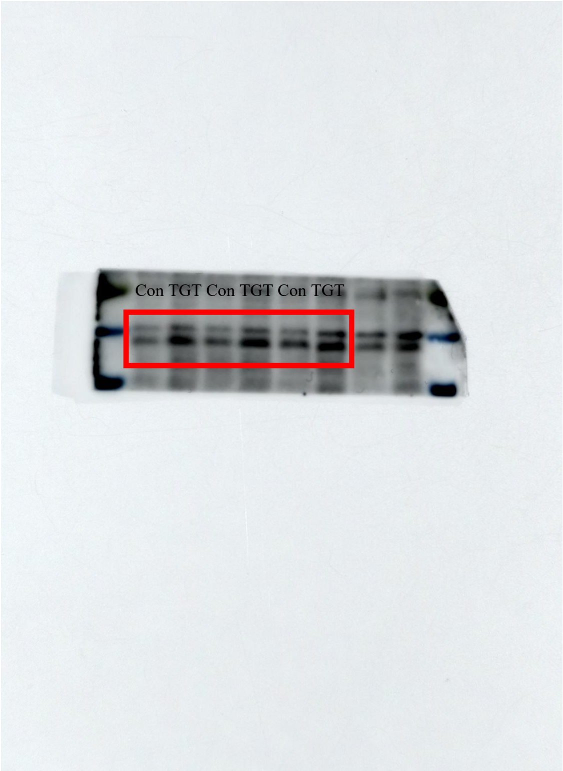

143B ACTIN

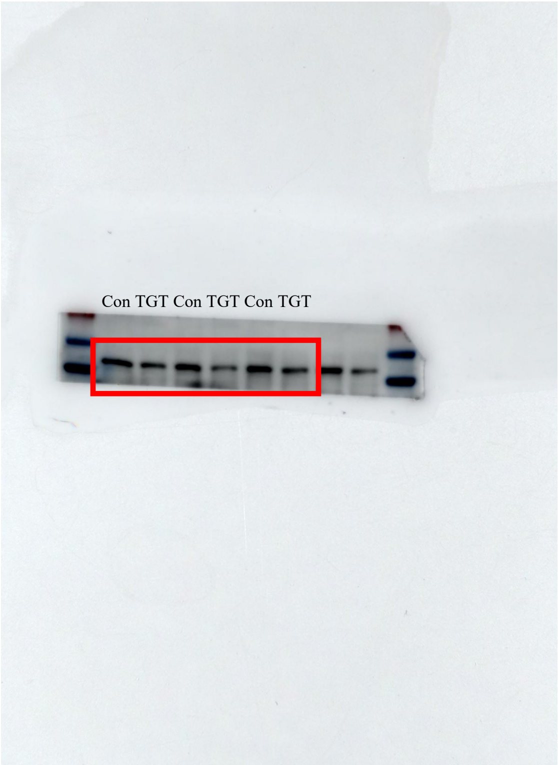

143B BCL2

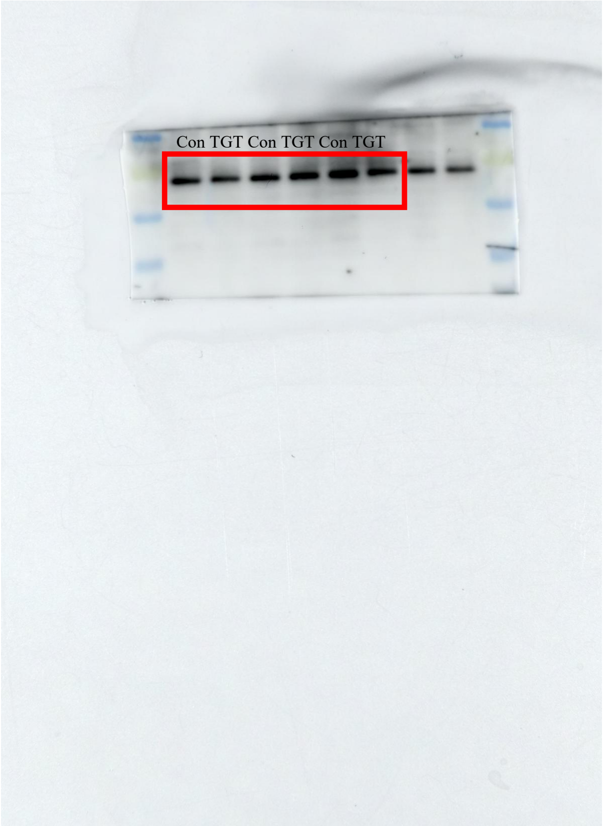

143B ACTIN

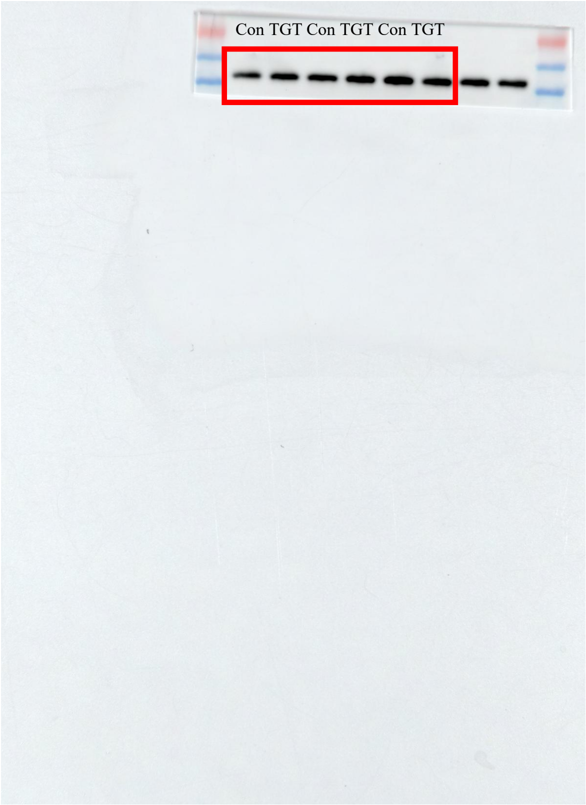

143B PCNA

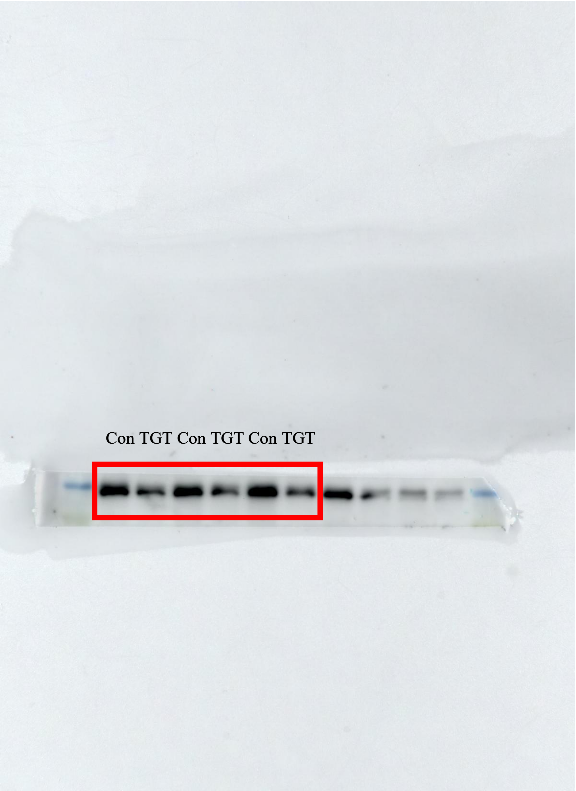

143B ACTIN

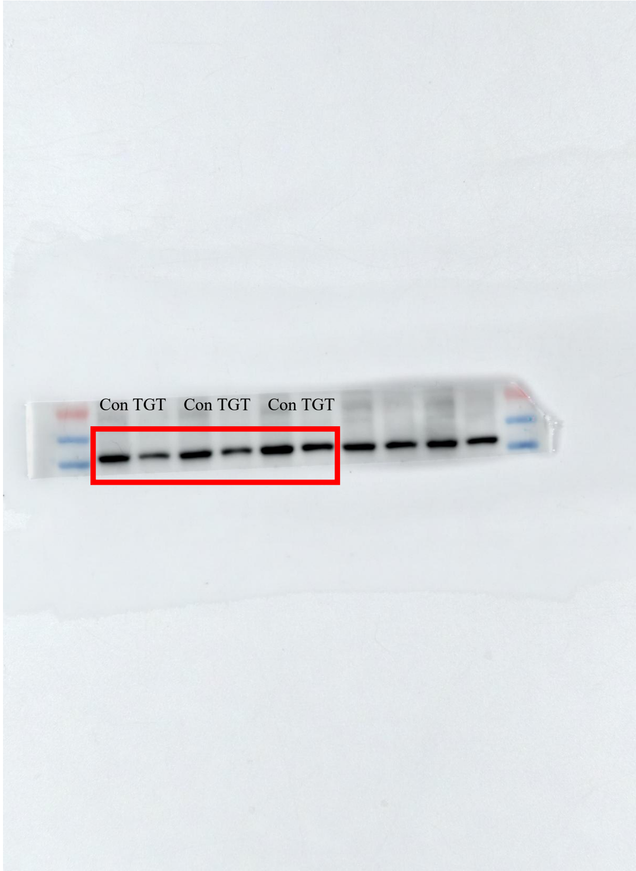

SAOS2 BAX

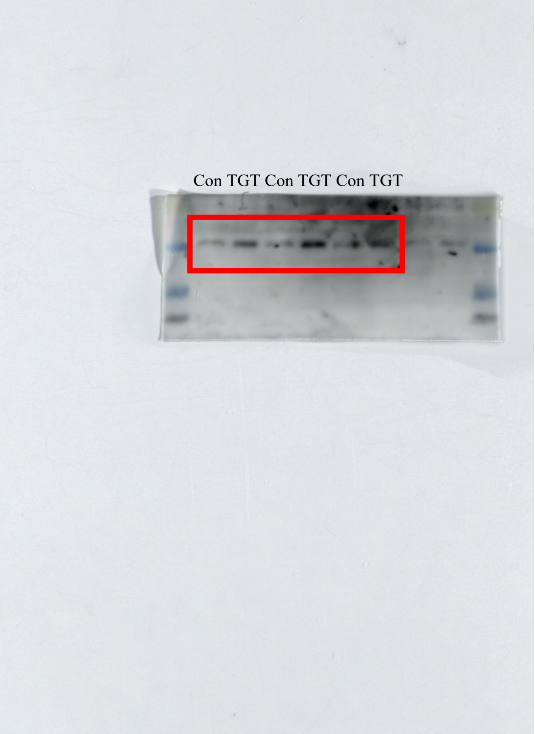

SAOS2 ACTIN

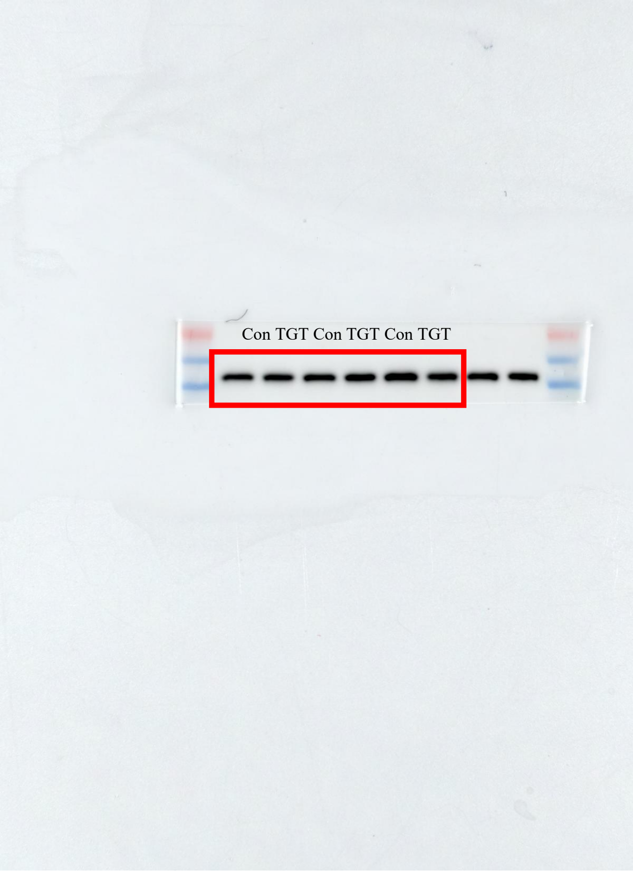

SAOS2 BCL2

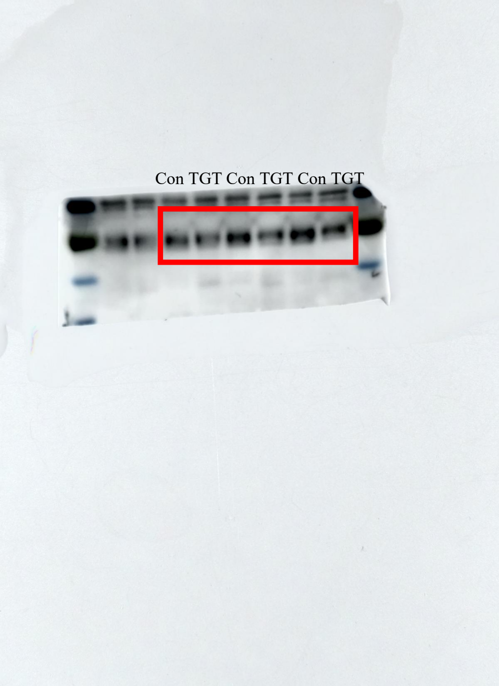

SAOS2 ACTIN

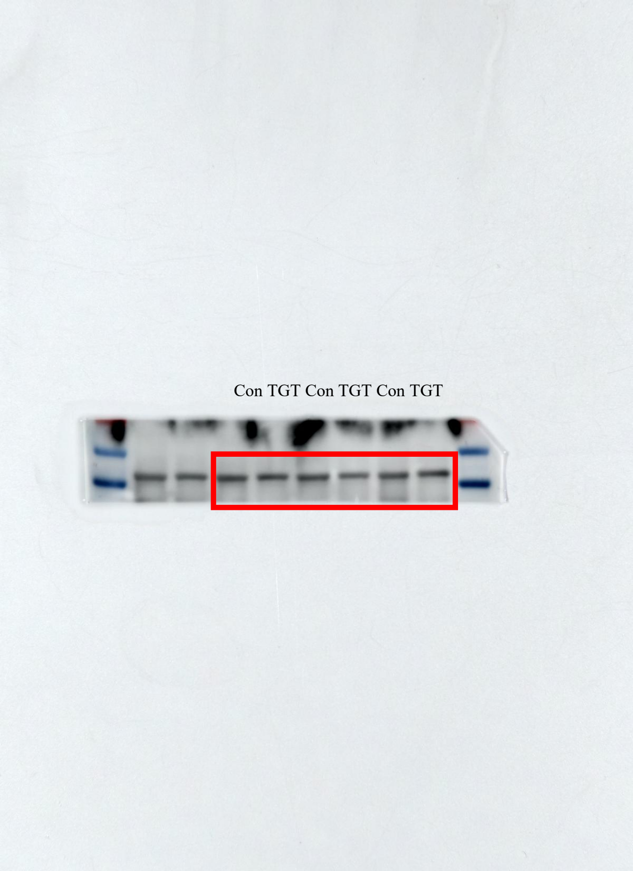

SAOS2 PCNA

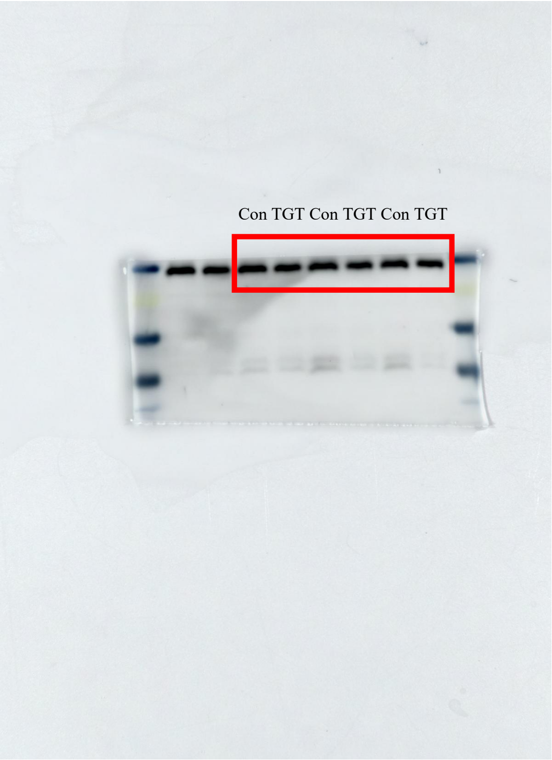

SAOS2 ACTIN

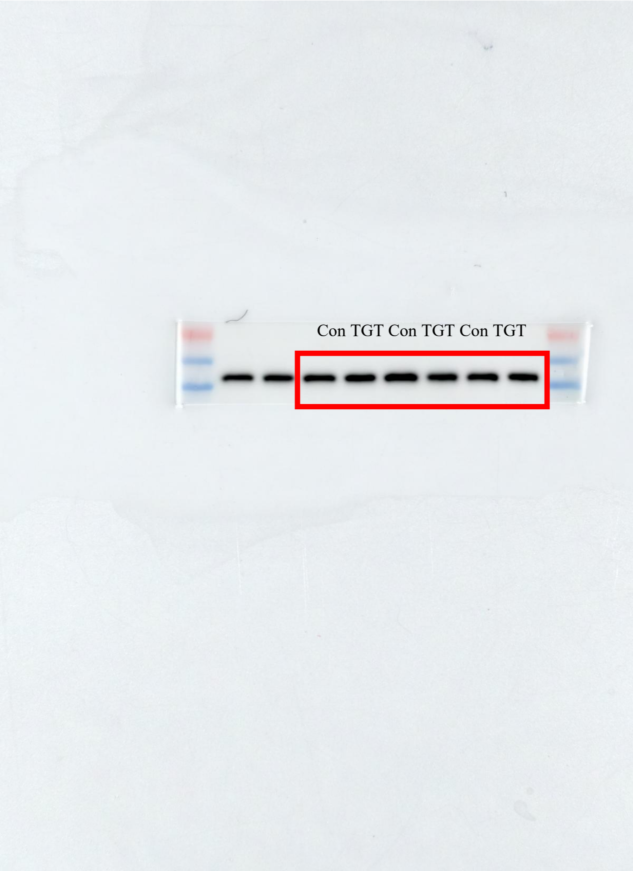

Supplement: Supplementary file 3 — Additional file 3. [file 12906_2024_4354_MOESM3_ESM.pdf]
